# Cation–Anion Synergy Enables Uniform and Stable SAMs for High‐Efficiency Perovskite/TOPCon Tandem Solar Cells

**DOI:** 10.1002/advs.202520822

**Published:** 2026-02-15

**Authors:** Haofan Ma, Xin Li, Huan Li, Jianmin Guan, Luyao Zheng, Jun Wu, Ziyu He, Yunyun Yu, Jungan Wang, Jie Yang, Xinyu Zhang, Meili Zhang, Yuheng Zeng, Menglei Xu, Zhiqin Ying, Xi Yang, Jichun Ye

**Affiliations:** ^1^ School of Materials Science and Engineering Shanghai University Shanghai P. R. China; ^2^ Zhejiang Provincial Engineering Research Center of Energy Optoelectronic Materials and Devices Ningbo Institute of Materials Technology and Engineering Chinese Academy of Sciences Ningbo P. R. China; ^3^ Zhejiang Jinko Solar Co., Ltd Jiaxing Zhejiang P. R. China; ^4^ Zhejiang Key Laboratory of Advanced Tandem Photovoltaic Technology Jiaxing Zhejiang P. R. China; ^5^ Yongjiang Laboratory Ningbo Zhejiang P. R. China

**Keywords:** cation–π interaction, deprotonation, self‐assembled monolayers, TOPCon tandem solar cells

## Abstract

Hole‐selective self‐assembled monolayers (SAMs) have recently boosted the efficiency of perovskite/silicon tandem solar cells (TSCs), but constructing dense, uniform, and particularly stable SAMs on textured surfaces remains challenging. Here, a cation–anion synergistic strategy is employed to suppress SAMs clustering and enhance molecular anchoring stability. The combined effects of cation–π interaction and phosphonic acid deprotonation enable the formation of high‐quality SAMs and perovskite absorbers, while simultaneously improving their interfacial contact at the buried interface. The resulting wide‐bandgap perovskite solar cells yielded a power conversion efficiency (PCE) of 23.04%, maintaining 84% of their initial efficiency after 1500 h of maximum power point (MPP) tracking under ISOS‐L‐1 protocol. Moreover, a 1 cm^2^ monolithic perovskite/silicon tandem solar cell based on textured tunnel oxide passivated contacts (TOPCon) delivered an impressive efficiency of 32.13%, representing the highest reported value to date for perovskite/TOPCon tandems.

## Graphical abstract

A cation‐anion synergistic strategy is developed to enhance self‐assembled monolayer (SAM) adsorption using guanidinium thiocyanate (GuaSCN). Guanidinium (Gua⁺) suppresses micelle aggregation, while thiocyanate (SCN−) promotes phosphonic acid deprotonation and strengthens ionic bonding to the substrate. The resulting perovskite solar cells (PSCs) and perovskite/tunnel oxide passivated contact (TOPCon) tandem devices achieve power conversion efficiencies (PCEs) of 23.04% and 32.13%, respectively.

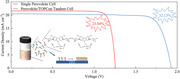

## 1 Introduction

Perovskite/silicon tandem solar cells (TSCs) have achieved power conversion efficiencies (PCEs) >33.7%, surpassing the Shockley−Queisser limit of any single‐junction solar cells [1, 2, 3, 4, 5]. Such progress is mainly driven by the broad implementation of organic SAMs in perovskite/silicon tandem architectures [1, 2, 3, 6]. However, perovskite/silicon TSCs still encounter challenges in self‐aggregation, uniformity, and anchoring stability of hole‐selective SAMs on rough texture surfaces in solution processing, which detrimentally affect their efficiency, scalability, and durability.

Several strategies have been explored to ensure uniform, dense SAM on rough surfaces, such as molecular design [7, 8], co‐solvent engineering [9], aiming at enhancing intermolecular conjugation and electron delocalization, and disassembling micelles, which facilitates better dispersion of SAMs in polar environments. However, molecule design is time‐intensive, and these methods primarily focus on modifying SAM itself, while largely neglecting the interfacial reaction between the SAM and the underlying substrate. Given that the formation of ultrathin SAMs proceeds via a condensation reaction between the P─OH bonds of the SAM molecules and the surface hydroxyl (─OH) groups of the substrate [10], co‐assembly of SAMs with distinct steric hindrances [11], nickel oxide/SAMs bilayers [12, 13], or substrate surface reconstruction engineering [13, 14, 15] have correspondingly proposed to provide more SAM molecules or to construct abundant OH groups for enabling more uniform and denser anchoring of SAMs on the substrates. Despite their promise, a certain proportion of SAMs remains weakly adsorbed via hydrogen bonding or without any binding mode due to the inadequate condensation reaction [16], which will cause the subsequent desorption of SAM from the substrate surface, ultimately leading to erratic interfacial properties. Therefore, achieving uniform, closely packed, and stable SAM anchoring on the substrate remains challenging [17].

Here, a cation‐anion synergist‒guanidinium thiocyanate (GuaSCN) is introduced into the [4‐(3,6‐Dimethyl‐9H‐carbazole‐9‐yl) butyl] phosphonic acid (Me‐4PACz) SAM solution to address the above issues. Specifically, the Gua^+^ cations, featuring a delocalized positive charge, form a strong cation–π interaction with the carbazole moiety of Me‐4PACz SAMs, disrupting micelle formation and increasing the dispersity of SAMs, which provides a fundamental prerequisite for obtaining denser SAM. Simultaneously, the SCN^−^ anions with a high base dissociation constant facilitate the deprotonation equilibrium of the phosphonic acid group, allowing to direct ionic bonding of SAMs with the substrate. Consequently, a high‐density, uniform, and very stable anchored Me‐4PACz SAM is obtained, which not only facilitates high‐quality perovskite film growth with reduced defects but also enables superior interface selective contact. As a result, single‐junction wide‐bandgap perovskite solar cells (PSCs) deliver an efficient PCE of 23.04% with decent stability (Over 84% efficiency retained after more than 1500 h of maximum power point (MPP) tracking, ISOS‐L‐1). When integrated on the textured tunnel oxide passivated contacts (TOPCon), a 1 cm^2^ monolithic perovskite/silicon tandem solar cell with a high efficiency of 32.13% is achieved.

## 2 Result and Discussion

**FIGURE 1:**
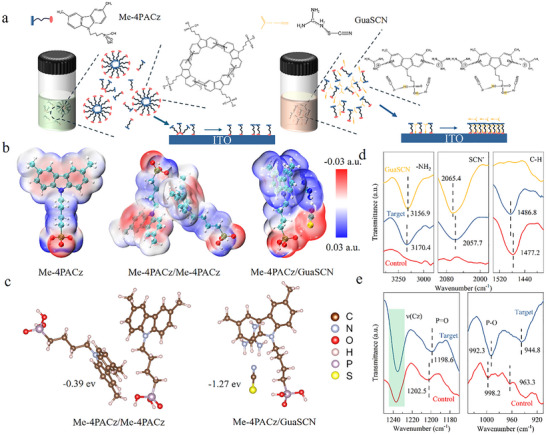
(a) Structural depiction of GuaSCN‐assisted micellar dispersion of Me‐4PACz. (b) Electrostatic potential (ESP) maps of Me‐4PACz, Me‐4PACz/Me‐4PACz, and Me‐4PACz/GuaSCN. (c) DFT‐calculated binding energies between Me‐4PACz and GuaSCN, as well as between two Me‐4PACz molecules. (d) FTIR‐ATR of Me‐4PACz /EtOH and Me‐4PACz (GuaSCN)/EtOH solutions. (e) FTIR of Me‐4PACz and Me‐4PACz (GuaSCN) films on ITO surface.

Figure 1a illustrates the mechanism by which GuaSCN facilitates the dispersion of Me‐4PACz micelles and facilitates their subsequent adsorption onto the ITO substrate. In ethanol, Me‐4PACz molecules tend to spontaneously aggregate into undesirable micelles due to strong π–π interactions among carbazole moieties, with the phosphonic acid groups exposed outwardly to the polar environment. Upon spin‐coating, these micelles exhibit limited interfacial contact with the hydroxyl‐rich ITO surface owing to their low surface‐to‐volume ratio, which compromises the binding efficiency of the phosphonic acid groups to the substrate. and ultimately suppressing self‐assembly into a uniform monolayer. The introduction of GuaSCN into the Me‐4PACz solution mitigates this aggregation. The Gua^+^ cations exhibit a unique Y‐aromatic character, where terminal atoms delocalize electrons via the central atom's orbitals, enabling strong cation−π interactions with carbazole units. These interactions effectively outcompete conventional π−π stacking, increasing the intermolecular spacing among carbazole groups and reducing associated undesirable micelle formation. Eventually, more Me‐4PACz molecules remain as monomers rather than micelle nanoparticles in solution, facilitating high‐density and uniform adsorption on the ITO surface. Simultaneously, the SCN^−^ ions are expected to facilitate the deprotonation equilibrium of the ─PO(OH)_2_ group in Me‐4PACz [18, 19]. Given the significantly higher base dissociation constant of SCN^−^ (pK_b_ ≈ 14.8) compared to the acid dissociation constants of the phosphonic acid protons (pK_a1_ = 2.5, pK_a2_ = 8.5), the phosphonic acid of Me‐4PACz will be partially deprotonated. This promotes direct iconic bonding with the ITO surface, enhancing interfacial binding strength beyond that achieved through hydrogen bonding. Furthermore, residual GuaSCN selectively accumulates atop the Me‐4PACz layer, serving as nucleation sites for perovskite growth while effectively passivating defects at the buried interface.

To elucidate the binding interactions between GuaSCN and Me‐4PACz, density functional theory (DFT) calculations were first employed. As depicted in Figure 1b, the electrostatic potential (ESP) map highlights an electron‐rich Lewis basic region on the Me‐4PACz carbazole moiety, which engages in strong cation−π conjugation and electronic coupling with Gua^+^. Consequently, the binding energy of the /Me‐4PACz (GuaSCN) complex (−1.27 eV) is markedly greater than that of the Me‐4PACz homodimer (−0.39 eV) (Figure 1c). These results demonstrate that Me‐4PACz preferentially associates with GuaSCN via cation−π interaction, facilitating micelle dissociation, enhancing molecular dispersion in solution [20]. Dynamic light scattering (DLS) measurements revealed that the micelle size of Me‐4PACz decreases from 350 to 80 nm upon adding GuaSCN (Figure S1). The critical micelle concentration (CMC) of Me‐4PACz also increases from 0.036 to 0.085 mg/mL after GuaSCN incorporation (Figure S2), confirming the enhanced dispersion effect of GuaSCN [21, 22]. Additionally, GuaSCN‐doped Me‐4PACz solution exhibits a significantly decreased static contact angle of 16.3° on the ITO substrate (Figure S3) and a more rapid decrease of dynamic contact angle (Figure S4), suggesting faster spreading and more efficient diffusion across the ITO surface, which further supports the improved dispersibility of Me‐4PACz in the presence of GuaSCN.

Fourier‐transform attenuated total reflectance infrared spectroscopy (FTIR‐ATR) was then employed to analyze the intermolecular interactions between GuaSCN and Me‐4PACz. As shown in Figure 1d, the signature peak of the benzene ring in the Me‐4PACz solution (denoted as “control”) at 1477.2 cm^−1^ exhibits a 9.6 cm^−1^ blueshift in the Me‐4PACz (GuaSCN) mixed solution (denoted as “target”). Similarly, the NH_2_ group in GuaSCN exhibits a 3.5 cm^−1^ shift to a higher wavenumber. This spectral shift confirms the presence of cation–π interactions between Me‐4PACz and GuaSCN [23, 24, 25]. In addition, the SCN^−^ vibrational peak exhibits a redshift of 7.7 cm^−1^, while the P─OH peaks in the target sample shift to higher wavenumbers from approximately 926.4 and 998.9 cm^−1^ (Figure S5) [26]. Moreover, the ─OH stretching vibrations observed in the control sample become negligible after the addition of GuaSCN (Figure S6). indicating the deprotonation interactions between GuaSCN and Me‐4PACz, which can be further confirmed by the lower pH value of the GuaSCN‐added SAM solution (Figure S7). This synergistic effect significantly enhances the dispersion of SAM molecules in solutions and facilitates their tight adsorption onto ITO.

NMR measurements further substantiate the strong interactions between GuaSCN and Me‐4PACz. As shown in Figure S8, upon introducing GuaSCN, the ^13^C signals associated with the unsaturated carbons of the Me‐4PACz carbazole ring shift downfield, whereas the carbon resonances of Gua^+^ exhibit an upfield shift, confirming that Gua^+^ engages strongly with the π‐conjugated carbazole ring and perturbs the local electron distribution. Consistent variations observed in the ^1^H spectra further corroborate this interaction (Figure S9). Additionally, the ^31^P resonance of Me‐4PACz undergoes an upfield shift after GuaSCN incorporation (Figure S10), indicating SCN^−^‐induced deprotonation of the phosphonic acid group. This deprotonation increases the electron density around the phosphorus center, thereby enhancing the nuclear shielding.

X‐ray photoelectron spectroscopy (XPS) reveals the variation of S and N elements on the ITO/Me‐4PACz surface after GuaSCN incorporation, confirming the successful integration of GuaSCN into Me‐4PACz (Figures S11 and S12). Figure S13 illustrates the Bonding between the SAM and the anchor groups on the ITO substrate. The O 1s spectrum of ITO reveals four distinct peaks at 530.35, 531.32, 532.24, and 533.27 eV, attributed to In/Sn‐O, oxygen vacancies, In─O─P/In─O─H and H_2_O [27] respectively. The target SAM exhibits a higher In─O─P/In─O─H peak area ratio (23.15%) compared to the control (15.64%), indicating improved molecular coverage [21]. Additionally, the P 2p spectrum of the target SAMs shows a decrease in the phosphorus binding energy (Figure S14), indicating that the PA groups in the target SAMs form a stronger tridentate anchoring interaction with the ITO surface [28, 29, 30]. We also employed Fourier‐transform infrared spectroscopy (FTIR) to analyze the interfacial chemical bonding characteristics (Figure 1e). The target sample exhibited stronger stretching vibrations compared with the control sample in both the P─O and P═O modes, with redshifts of 7.6 and 3.9 cm^−1^ [22, 30, 31]. Furthermore, the carbazole ring (ν(Cz)) in the target sample displayed more intense and sharper vibrational peaks, indicating enhanced binding between Me‐4PACz and the ITO surface [32].

We explored the effect of GuaSCN additives on the surface energy level structure of SAMs. Atomic force microscopy (AFM) was employed to examine how GuaSCN incorporation influences the surface morphology of ITO/Me‐4PACz films. The non‐uniform adsorption of Me‐4PACz on ITO led to an increase in surface roughness from 2.16 to 2.28 nm. After the incorporation of GuaSCN, the surface roughness decreased to 2.00 nm (Figure S15). Kelvin Probe Force Microscopy (KPFM) was used to evaluate the surface potential of SAMs coated on ITO substrates, as shown in Figure S16, with ITO/Me‐4PACz (GuaSCN)exhibiting a significantly lower contact potential difference (CPD) (−505 mV) compared to ITO/Me‐4PACz (−235 mV), indicating increased surface work function of ITO/Me‐4PACz (GuaSCN) [33]. Simultaneously, a more homogeneous surface distribution of ITO/Me‐4PACz (GuaSCN) is observed, which benefits from the uniform assembly of SAM (Figure S17). Ultraviolet photoelectron spectroscopy (UPS) measurements (Figure S18) identified the energy levels of ITO/Me‐4PACz and ITO/Me‐4PACz (GuaSCN). These results demonstrate that after treatment with Me‐4PACz and Me‐4PACz (GuaSCN), the work function of ITO increased to 4.91 and 5.02 eV, respectively, consistent with the KPFM results, thereby enhancing hole selectivity and promoting the interfacial hole extraction process [34, 35]. Conductivity measurements further confirmed the enhanced hole transfer after GuaSCN modification (Figure S19). Moreover, as shown in Figure S20, the incorporation of GuaSCN does not diminish the optical properties of the SAMs.

**FIGURE 2:**
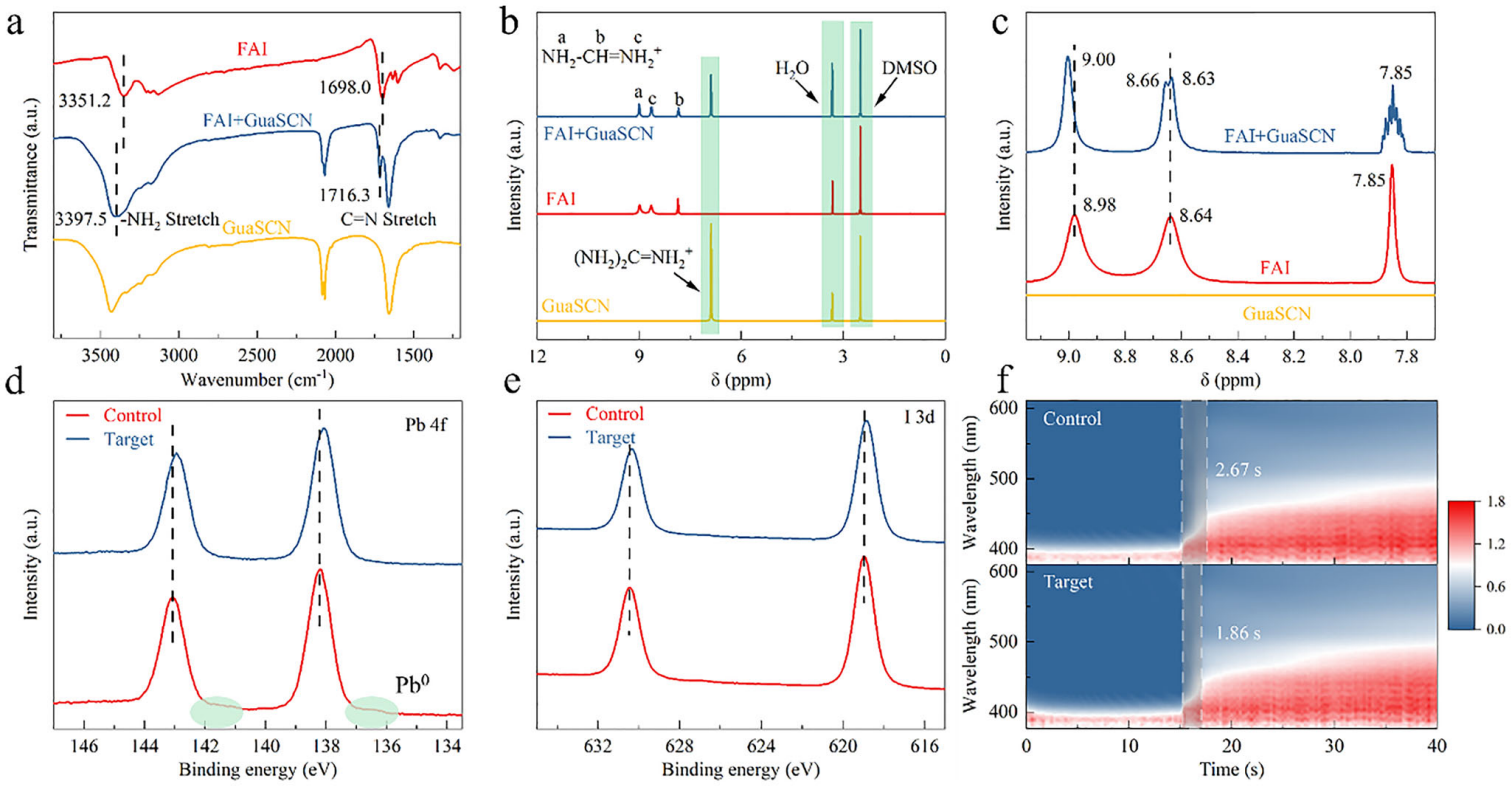
(a) FTIR spectra of FAI, GuaSCN, and their mixture (FAI + GuaSCN). (b) ^1^H‐NMR spectra of FAI, GuaSCN, and FAI + GuaSCN hybrid and (c) the corresponding local enlarged spectra. The XPS spectra for the buried interface of perovskite films of Pb 4f (d) and I 3d (e) for comparing samples with and without GuaSCN treatment. (f) In situ UV–vis absorption contour plots during perovskite precursors spin‐coating on control and target SAM‐modified substrates.

The interaction between GuaSCN and precursor solutes significantly governs perovskite nucleation and crystal growth. As characterized by FTIR in Figure 2a, the FA^+^ skeletal vibration and −NH_2_ stretching vibration of pure FAI appear at 1698.0 and 3351.2 cm^−1^. When mixed with GuaSCN, these characteristic peaks shift significantly to higher wavenumbers, indicative of hydrogen bond interactions [36]. For the PbI_2_ sample, the −SCN stretching mode shifts to a lower wavenumber due to chelation interaction of −SCN with the under‐coordinated Pb^2+^ (Figure S21) [37]. ^1^H nuclear magnetic resonance (^1^H‐NMR) analysis, shown in Figure 2b,c, reveals that after mixing FAI with GuaSCN, the proton signal of the −NH_2_ group in FAI upshifts by 0.02 ppm, confirming hydrogen bonding with each other [38]. XPS analysis was carried out on the peeled perovskite film to determine the effectiveness of defect passivation at the buried interface (Figure S22) [39]. As depicted in Figure S23, hydrogen bonding between FA^+^ and Gua^+^ is evidenced by a shift in the N 1s characteristic peak to higher binding energy. Additionally, the Pb 4f peaks at 138.2 and 143.1 eV shift to lower binding energies after GuaSCN treatment compared with the control sample (Figure 2d), suggesting a reduced oxidation state of Pb^2+^, which is associated with the formation of a complex between −SCN and under‐coordinated [PbI_6_]^4−^ [40]. A similar trend is observed in the I 3d region (Figure 2e) [41, 42, 43]. These results suggest that GuaSCN strongly interacts with the perovskite precursors, conducive to achieving a distinct crystallization mechanism and a thin‐film formation process.

To validate our hypothesis, contact angle measurements with perovskite inks were performed (Figure S24). Compared to the pristine ITO/Me‐4PACz surface, which exhibits a perovskite ink contact angle of 38.4°, the introduction of GuaSCN significantly reduces the contact angle to 6.6°, attributing to the increased surface free energy induced by GuaSCN (Figure S25 and Table S1), which in turn facilitates favorable nucleation during film formation and promotes more uniform perovskite coverage [44]. In‐situ ultraviolet‐visible (UV–vis) spectroscopy during antisolvent bathing was further conducted to elucidate the initial crystallization dynamics (Figure 2f). The perovskite films based on Me‐4PACz were denoted as the control, while those based on Me‐4PACz (GuaSCN) were designated as the target. Prior to the antisolvent dipping process, the perovskite film exhibits low absorption intensity. Upon antisolvent application, a marked increase in absorption is observed after 15 s, indicating the onset of crystallization. The control group displays a delayed increase in absorption intensity; this effect is likely caused by the non‐uniform adsorption and hydrophobic surface of Me‐4PACz, which limits nucleation of the perovskite layer. In contrast, GuaSCN modification leads to a more rapid rise in absorption intensity, reflecting enhanced heterogeneous nucleation and earlier initiation of perovskite crystallization. Moreover, we extracted the time‐resolved absorbance evolution at 490 nm, as shown in Figure S26, the absorbance of the GuaSCN‐modified precursor rises slightly earlier and reaches its maximum sooner than the control. The hydrogen bonding between Gua^+^ and FA^+^, combined with the coordination interactions between −SCN and undercoordinated Pb^2+^ ions, provides buried nucleation sites for early‐stage perovskite formation, thus promoting the crystallization process.

**FIGURE 3:**
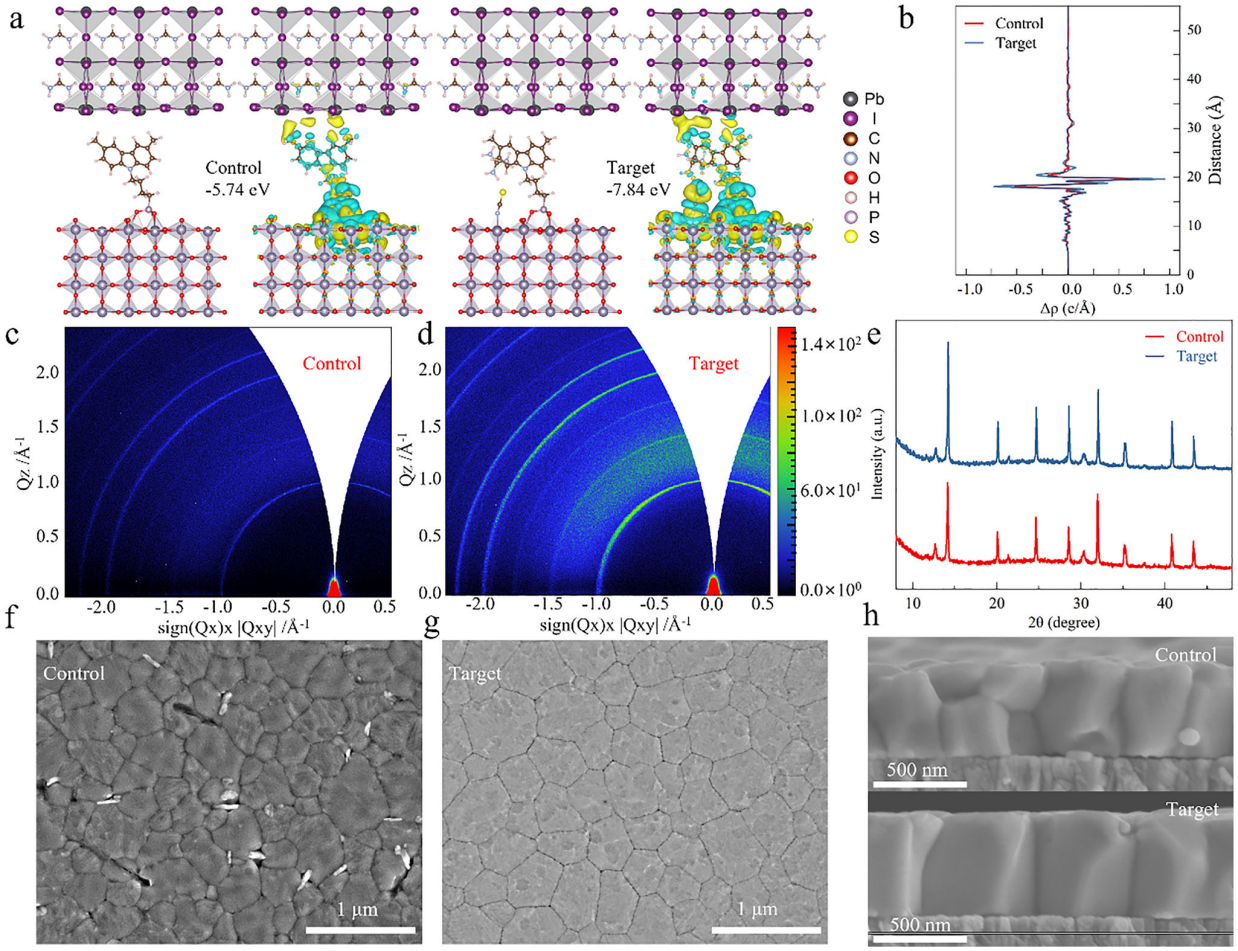
(a) The different charge densities of ITO/Me‐4PACz/PVK and ITO/Me‐4PACz+GuaSCN/PVK. (b) Charge density difference projected along the *z‐*axis. GIWAXS patterns of the buried interface of perovskite films on (c) Me‐4PACz and (d) Me‐4PACz (GuaSCN). (e) XRD patterns of perovskite films on Me‐4PACz and Me‐4PACz (GuaSCN). Bottom‐viewed SEM images (f) without and (g) with GuaSCN‐treated perovskite films. (h) Cross‐sectional SEM images without and with GuaSCN‐treated perovskite films.

We performed DFT calculations to explore the optimized configurations of two heterojunctions: ITO/Me‐4PACz/PVK and ITO/Me‐4PACz+GuaSCN/PVK. A vacuum region of approximately 14 Å was initially inserted at the ITO/PVK interface, and the SAM molecules were placed within this vacuum region for full structural relaxation (Figures S27 and S28). As illustrated in Figure 3a, the Me‐4PACz+GuaSCN heterojunction exhibits a significantly higher binding energy (−7.84 eV) compared to the Me‐4PACz‐only system (−5.74 eV), indicating stronger interfacial interactions. Furthermore, the differential charge density and its integrated profile along the vertical axis reveal a more pronounced charge transfer in the Me‐4PACz+GuaSCN system compared to Me‐4PACz alone (Figure 3b), implying that the presence of GuaSCN improves interfacial charge transport and modulates the local electronic structure at the interface [18]. Additionally, SCN^−^ can simultaneously coordinate with both Gua^+^ and ITO at the interface, while strong cation−π interactions are established between Gua^+^ and the carbazole moiety of the anchored Me‐4PACz. This bridging coordination strengthens electronic coupling across the interface and improves the structural integrity of the SAM layer at the buried interface [45].

To evaluate the consequence of GuaSCN incorporation on perovskite films, a thorough analysis of their crystal structure and morphology was performed. Grazing‐incidence wide‐angle X‐ray scattering (GIWAXS) was employed to investigate the crystallization behavior at the buried interface of the films before and after GuaSCN treatment. As shown in Figure 3c,d, both films exhibit weak intensity of PbI_2_ at q_z_ = 0.9 Å^−1^. However, the GuaSCN‐based film shows higher radial integration intensities at (100), (110), and (210), and the q_z_ profiles extracted from the corresponding 2D GIWAXS pattern demonstrate that the intensity ratio of (100)/(110) for the target film is 1.48, notably higher than the control film (1.13), confirming that GuaSCN modification promotes enhanced vertical orientation of the perovskite crystals. (Figure S29). Furthermore, both the q_z_ profiles and the 2θ patterns reveal improved crystallinity in the perovskite films (Figure S30), where the (100)/PbI_2_ intensity ratio is markedly higher than that of the control sample (Tables S4 and S5). These results further confirm the beneficial role of GuaSCN in promoting high‐quality perovskite crystallization. X‐ray diffraction (XRD) characterization revealed enhanced crystal quality of the perovskite film following GuaSCN incorporation, as evidenced by the increased (100)/PbI_2_ intensity ratio and reduced FWHM of the (100) peak (Figure 3e; Figure S31) [38].

Scanning electron microscopy (SEM) was then used to examine the surface morphology of the GuaSCN‐modified perovskite films at the buried interface. Figure 3f,g reveal that the interface between the Me‐4PACz layer and the perovskite film contains nanoscale voids, attributed to its hydrophobicity and poor wetting, along with noticeable residual PbI_2_. After GuaSCN incorporation, the bottom surface displays a denser and smoother film morphology, with significantly reduced PbI_2_ residue. Additionally, top‐interface SEM images show that after GuaSCN modification, the mean grain size of the perovskite film increases from 373 to 519 nm (Figure S32). The SEM cross‐sectional image reveals that the perovskite grains in the control sample grow in a random and disordered manner and delaminate with Me‐4PACz. In contrast, the perovskite film modified with GuaSCN exhibits enhanced crystallinity, characterized by significantly enlarged grains that are vertically aligned with respect to the substrate (Figure 3h), indicative of oriented crystal growth. Moreover, the adhesion at the contact between the substrate and the perovskite layer is significantly enhanced, with no signs of delamination observed, which benefits the release of the residual stress (Figure S33). Moreover, characterization confirmed that the control film possessed a roughness of 18.4 nm, whereas the GuaSCN‐modified perovskite film exhibits a roughness of 12.8 nm (Figure S34). The results demonstrate that a smooth substrate significantly contributes to the development of high‐quality perovskite films, and lower roughness results in better interlayer contact between the perovskite film and the electron transport layer, promoting more efficient electron transfer and extraction [46].

**FIGURE 4:**
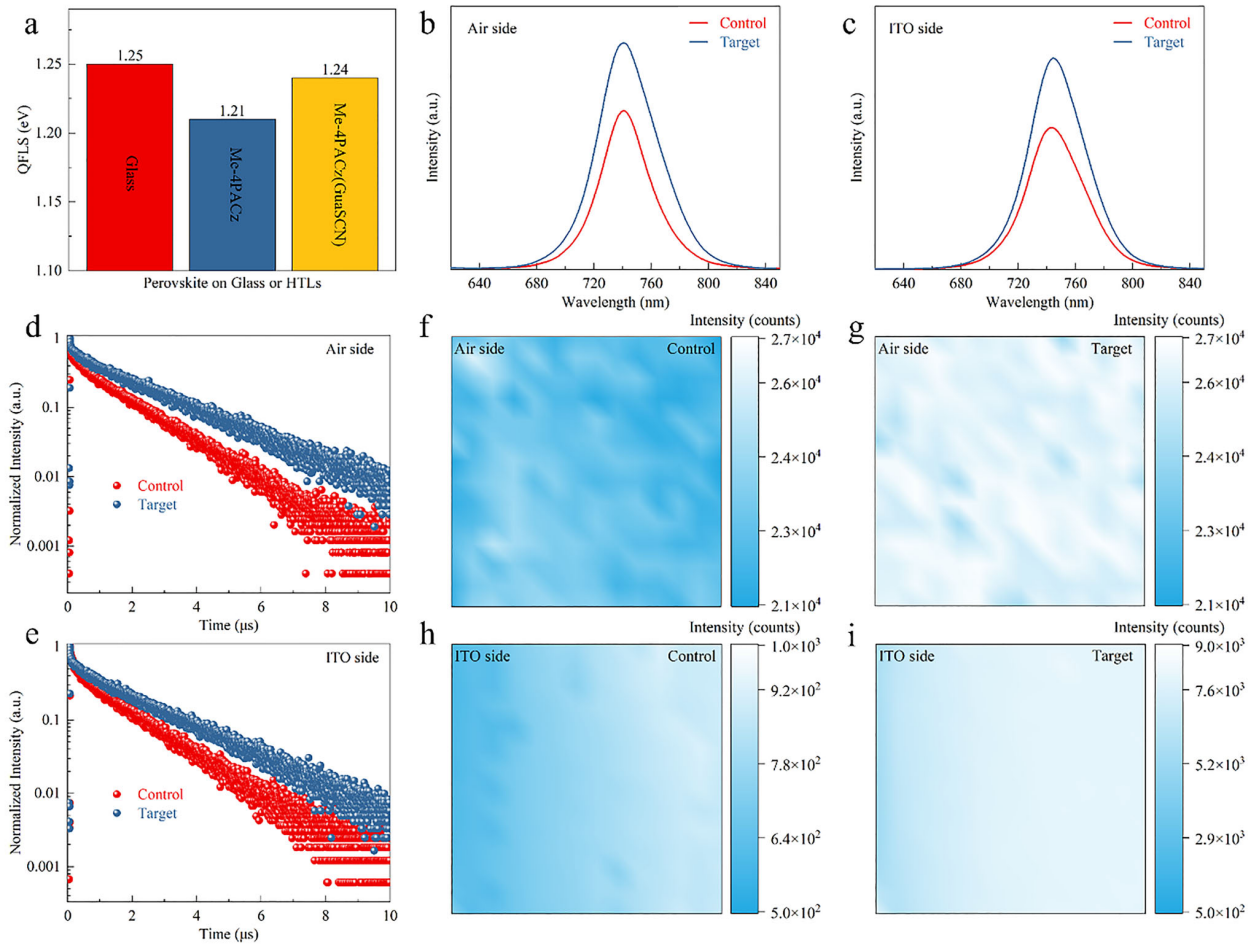
(a) The calculated QFLS of perovskite films on Glass, control, and target films. (b–e) SSPL and TRPL spectra of perovskite films with control and target interface. PL mapping images of the perovskite films at the control interfaces (f,h) and target interfaces (g,i) under excitation from different directions.

To gain insights into how the GuaSCN incorporation would impact the defect passivation and charge carrier dynamics in halide perovskite, absolute photoluminescence (PL) measurements for determining the internal quasi‐Fermi level splitting (QFLS) of perovskite films was performed (Figure 4a). The QFLS of the perovskite directly deposited on the glass substrate is 1.25 eV, whereas that of the control sample was 1.21 eV, indicating substantial recombination via defect states at the Me‐4PACz/perovskite interface. In contrast, the target sample exhibits an increased QFLS of 1.24 eV, confirming that GuaSCN effectively suppresses non‐radiative recombination through chemical passivation and energy band optimization, which is predicted to increase the device's *V_OC_* and FF. To further validate these findings, measurements of steady‐state (SSPL) and time‐resolved photoluminescence (TRPL) were conducted on both the air and ITO sides of the films. After GuaSCN incorporation, a notable enhancement in steady‐state PL intensity was observed, with improvements of 1.42 and 1.48 times on the air and ITO sides, respectively (Figure 4b,c). Previous studies indicate that carbazole‐based SAMs do not suppress the PL intensity of perovskite films [47], as they help suppress trap‐assisted non‐radiative recombination. This result suggests that GuaSCN effectively passivates both the buried interface and intrinsic defects in the perovskite layer. TRPL analysis (Figure 4d,e) reveals that the early decay time, typically associated with interface charge transfer processes, exhibits a much faster initial decay within the first 10 ns when excited from the ITO side. As shown in Figure S35, we calculated the differential lifetime to directly extract the transient decay time [37]. Compared to the control, the target film displayed a significantly quicker initial rise in lifetime, suggesting that GuaSCN incorporation facilitates more efficient carrier transfer at the buried interface. Double‐exponential fitting (Tables S2 and S3) reveals that the effective carrier lifetime increases from 1338.68 ns in the control film to 1834.23 ns in the target film, representing a 1.37‐fold enhancement. This suggests that defect passivation at the bottom perovskite surface is effective, therefore reducing interface recombination. When illuminated from the perovskite side, the target perovskite film's lifetime increased by 1.57 times compared to the control, indicating successful passivation of intrinsic defects within the perovskite film. Additionally, we employed PL mapping from both sides to assess the spatial uniformity of the perovskite films (Figure 4g–i). Over a 50 × 50 µm2 measurement area, the PL intensity of the target film is markedly higher and more evenly distributed on both the perovskite and ITO interfaces, whereas the control film shows weaker emission and poorer uniformity, particularly on the ITO side, further confirming that GuaSCN effectively passivates interfacial defects at the buried interface.

**FIGURE 5:**
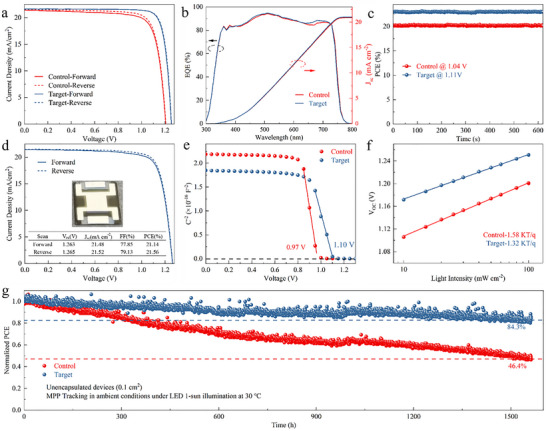
(a) *J*−*V* curves of the champion Me‐4PACz and Me‐4PACz (GuaSCN) based single‐junction PSCs. (b) EQE spectra of both devices with integrated short‐circuit current densities (*J_SC_*). (c) SPO performance devices. (d) *J*−*V* curve of a 1.21 cm^2^ aperture area champion PSC. (e,f) Mott–Schottky plots and light‐intensity‐dependent of *V_OC_* of the Me‐4PACz and Me‐4PACz (GuaSCN) based devices. (g) The MPP tracking of Unencapsulated PSCs.

We further constructed inverted perovskite solar cells (PSCs) with a configuration of ITO/SAMs/ (Cs_0.05_ (FA_0.77_MA_0.23_)_0.95_Pb(I_0.77_Br_0.23_)_3_)/C_60_/bathocuproine (BCP)/Ag to assess device performance (Figure S36). Device performance was evaluated under varying GuaSCN modification concentrations to identify the most effective level (Figure S37). The Me‐4PACz (GuaSCN)‐based devices outperformed the Me‐4PACz devices, primarily due to simultaneous enhancements in *V*_OC_ and FF (Figure S38). Figure 5a presents the *J*−*V* characteristics of the champion inverted PSCs employing different HTLs. The champion device with Me‐4PACz exhibits a power conversion efficiency (PCE) of 20.70%, with a *V*_OC_ of 1.208 V and an FF of 79.64%. In contrast, the Me‐4PACz (GuaSCN) device achieved a maximum efficiency of 23.04%, with a *V*_OC_ of 1.256 V and an FF of 84.84% under reverse scan. Moreover, the processed PSCs feature negligible hysteresis, which can be attributed to the enhanced carrier transport facilitated by the GuaSCN‐modified substrate. The external quantum efficiency (EQE) of the champion device with Me‐4PACz (GuaSCN) yields a comprehensive *J*_SC_ value of 21.00 mA cm^−2^ (Figure 5b), and the steady‐state PCE is 22.99% (Figure 5c). To evaluate scalability, we further fabricated perovskite solar cells with a 1.21 cm^2^ aperture area (Figure 5d). The optimal target device achieved a PCE of 21.56%, with a *V*_OC_ of 1.265 V and an FF of 79.13% under reverse scanning. Furthermore, this cation−anion cooperative strategy demonstrates strong extensibility, enabling improved dispersion of SAMs containing various functional head groups in solution and facilitating the fabrication of high‐performance PSCs. Upon mixing GuaSCN with MeO‐2PACz (Figure S39), the micelle size is reduced from 378 to 58 nm, resulting in an efficiency enhancement from 21.23% to 22.37%. In the case of 4PADCB (Figure S40), the micelle size similarly decreases from 512 to 57 nm, with the device efficiency increasing from 21.22% to 22.61%. Both types of GuaSCN‐incorporated devices additionally exhibit significantly reduced hysteresis (Table S6). Moreover, GuaSCN also delivers pronounced performance enhancements in perovskite solar cells with different compositions and bandgaps (Figure S41), further confirming the broad applicability and universality of this strategy. These findings substantiate the effectiveness of GuaSCN in improving SAM micelle characteristics and boosting PSC performance.

Mott‐Schottky plots were utilized to support the improvement in both *V_OC_* and FF. Figure 5e illustrates that the built‐in potential (V_bi_) of the Me‐4PACz (GuaSCN)‐based device increases to 1.10 V, compared to 0.97 V for the Me‐4PACz‐based device, indicating improved carrier separation and transport efficiency. The relationship between light intensity and *V_OC_* was analyzed to further explore the charge recombination process. Following GuaSCN modification, the ideality factor (n) decreases to 1.32 (Figure 5f), suggesting suppression of trap‐assisted non‐radiative recombination. This improvement is attributed to the effective passivation effect of GuaSCN, thereby contributing to enhanced FF and *V*_OC_. Interfacial charge transfer was evaluated using electrochemical impedance spectroscopy (EIS), with Nyquist plot analysis (Figure S41) showing an increase in recombination resistance (R_rec_) after GuaSCN incorporation, further confirming reduced charge recombination. Furthermore, space‐charge limited current (SCLC) measurements were used to quantify the trap‐state density (N_trap_) in an ITO/SAMs/perovskite/spiro‐OMeTAD/Au device configuration. After the GuaSCN incorporation, the N_trap_ of the PSCs shows a significant decrease from 2.76 × 10^15^ to 1.94 × 10^15^ cm^−3^ (Figure S42), indicating a decrease in defect‐related recombination. This reduction in trap states also extends the carrier recombination lifetime, as evidenced by the slower photovoltage decay observed in transient photovoltage (TPV) measurements (Figure S43). Collectively, the GuaSCN‐treated devices demonstrate suppressed defect‐mediated recombination and enhanced charge carrier collection, ultimately leading to a substantially improved device performance.

To accurately evaluate device performance, we e conducted maximum power point tracking (MPPT) to test the stability of unencapsulated devices under continuous one‐sun illumination (AM1.5, 100 mW cm^−2^) at 30 °C and 40% RH (ISOS‐L‐1). As depicted in Figure 5g, after over 1500 h of continuous illumination, the target device preserves 84% of its original PCE, in contrast to just 46% retained by the control device. Based on the ISOS‐L‐2 protocol, we further conducted MPPT testing at 65 °C (Figure S44). The GuaSCN‐treated device demonstrates enhanced stability over 500 h compared to the Me‐4PACz‐based device. These results further demonstrate that the GuaSCN modification strategy effectively enhances the coverage and uniformity of the SAMs, and it markedly enhances the operational stability of perovskite solar cells.

**FIGURE 6:**
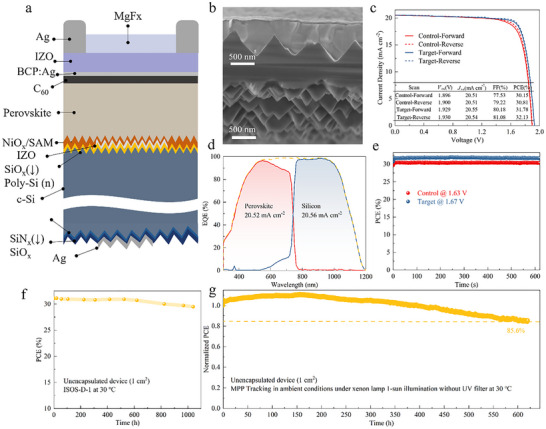
(a) Device structure schematic of the perovskite/TOPCon silicon tandem device. (b) Cross‐sectional SEM image of the tandem device. (c) *J*−*V* characteristics of the best‐performing device. (d) EQE spectrum of the perovskite/silicon tandem device. (e) SPO under continuous 1‐sun illumination. (f) Shelf stability of the unencapsulated GuaSCN‐based perovskite/ TOPCon silicon tandem device in N_2_. (g) The MPP tracking of the unencapsulated GuaSCN‐based tandem device.

Finally, leveraging the excellent performance of the improved SAMs on wide‐bandgap perovskites and their potential in conformal coating, we evaluated their applicability for integration with TOPCon silicon bottom cells in tandem devices (Figure 6a). Cross‐sectional SEM imaging (Figure 6b; Figure S45) confirms that the modified SAMs contribute to the formation of a uniform and well‐covered perovskite layer on the silicon substrate. The resulting tandem device, incorporating the enhanced SAMs, achieved an impressive PCE of 32.13%, with an FF of 81.08%, a *V*_OC_ of 1.930 V, and a *J*_SC_ of 20.54 mA cm^−2^, outperforming the control device (Figure 6c). The integrated *J*_SC_s values derived from the EQE spectra are 20.52 and 20.56 mA cm^−2^, respectively (Figure 6d). Additionally, by tracking the maximum power point for 10 min, the target tandem device achieves a steady‐state power output (SPO) efficiency of 31.7%, whereas the control device reaches 30.4% (Figure 6e), consistent with the *J−V* results. We obtained a certified efficiency of 31.81% under reverse scan with a steady‐state efficiency of 31.72% for the GuaSCN‐modified tandem device (Figure S46), which ranks among the highest efficiencies reported so far for monolithic perovskite/TOPCon tandem devices (Table S7). Shelf stability of the GuaSCN‐based tandem device was assessed using the ISOS‐D‐1 protocol, as illustrated in Figure 6f. After 1040 h at 30 °C and 10% relative humidity, the unencapsulated tandem device retained 94.8% of its initial PCE (31.1%). Furthermore, under continuous xenon lamp without a UV filter under 1‐sun illumination (ISOS‐L‐1), after 620 h, the unencapsulated GuaSCN‐based tandem maintained 85.6% of its initial PCE (30.87%) (Figure 5g).

## 3 Conclusion

In summary, a cation‐anion synergistic strategy is employed. The strong cation−π interaction induced by Gua^+^ cations significantly enhances the dispersion of SAMs in solution, while SCN^−^ anions promote the deprotonation equilibrium of the phosphonic acid group, synergistically enabling a uniform, dense, and stable SAM anchoring. Consequently, the 1.68 eV bandgap single‐junction perovskite solar cells delivered a PCE of 23.04% accompanied by excellent operational stability (maintaining 84% of its initial efficiency after 1500 h of device operation, ISOS‐L‐1 protocol). Notably, applied to textured perovskite/TOPCon tandem cells, this strategy enabled an impressive PCE of 32.13%.

## 4 Experimental Section

### 4.1 Materials

All chemicals and reagents were used as received from commercial sources. Formamidinium iodide (FAI) and Methylammonium bromide (MABr) were purchased from Great Cell Solar Materials, while cesium iodide (CsI), Me‐4PACz, guanidinium thiocyanate (GuaSCN), and anhydrous solvents (DMF, DMSO, and chlorobenzene) were obtained from Sigma–Aldrich. Lead (II) iodide (PbI_2_), lead (II) bromide (PbBr_2_), and phenylethyl ammonium diiodide (PDAI_2_, 99.5%) and [2‐(3,6‐dimethoxy‐9H‐carbazol‐9‐yl) ethyl] phosphonic acid (MeO‐2PACz) were sourced from TCI. C_60_ (99.5%) and bathocuproine (BCP, 99%) were provided by Taiwan Lumtec Corp. (4‐(7H‐dibenzo[c,g]carbazol‐7yl) butyl) phosphonic acid (4PADCB) was supplied by Suzhou LiWei Tech Co., Ltd. Pre‐patterned ITO glass substrates were acquired from Advanced Election Technology Co., Ltd., and metal electrode masks were custom fabricated by Shenzhen Rigorous Technology Co., Ltd.

### 4.2 Device Fabrication of the Perovskite Solar Cells

The ITO glass substrates were cleaned sequentially for 15 min each with conductive glass detergent, deionized water, acetone, and anhydrous ethanol. Afterward, the cleaned substrates were treated with UV‐ozone for 15 min to introduce hydroxyl groups on the surface. All of the next steps were performed in a N_2_‐filled glovebox (O_2_ < 0.01 ppm, H_2_O < 0.01 ppm). The substrates were spin‐coated with the Me‐4PACz and Me‐4PACz (GuaSCN) mixture. (To prepare a mixed Me‐4PACz (GuaSCN) solution, a 1 mg ml^−1^ Me‐4PACz solution and a 0.5 mg ml^−1^ GuaSCN were prepared in ethanol and stirred for 30 min). Next, 200 µL of this mixed solution was statically (with the rest time of ∼30 s) spin‐coated on the ITO substrate (4000 rpm, 30 s). Afterward, the ITO substrates were annealed at 100 °C for 10 min. CsI, FAI, MABr, PbBr_2_, and PbI_2_ were completely dissolved in 1 mL of a mixed solvent composed of DMF and DMSO in a 4:1 volume ratio, corresponding to the composition Cs_0.05_(FA_0.77_MA_0.23_)_0.95_Pb(I_0.77_Br_0_._23_)_3_ to obtain a 1.35 m perovskite precursor of 1.68 eV. CsI, FAI, PbI_2_, and PbBr_2_ were completely dissolved in 1 mL of a mixed solvent composed of DMF and DMSO in a 3:1 volume ratio, corresponding to the composition Cs_0.17_FA_0.83_Pb(I_0.83_Br_0.17_)_3_, to obtain a 1.6 m MA‐free perovskite precursor with a bandgap of 1.68 eV. CsI, FAI, MABr, PbI_2_, and PbBr_2_ were completely dissolved in 1 mL of a mixed solvent composed of DMF and DMSO in a 4:1 volume ratio, corresponding to the composition Cs_0.05_(FA_0.98_MA_0.02_)_0.95_Pb(I_0.98_Br_0.02_)_3_, to obtain a 1.5 m perovskite precursor with a bandgap of 1.55 eV. All solutions were filtered using 0.22 µm PTFE filters prior to use. To fabricate the perovskite layer, substrates were spin‐coated at 4000 rpm for 40 s, and the acceleration is 4000 rpm. During the final 15 s of spinning, 250 µL of chlorobenzene was dropped onto the rotating substrate. Perovskite films were annealed at 110 °C for 10 min, followed by spin‐coating of 1.5 mg mL^−1^ PDAI_2_ in isopropanol at 3000 rpm for 30 s and annealing at 105 °C for 5 min. Next, 20 nm of C_60_ and 6 nm of BCP were deposited via thermal evaporation under high vacuum (<5 × 10^−5^ Torr) at deposition rates of 0.2 and 1.0 Å s^−1^, respectively. Finally, thermally evaporated on top of the BCP layer at a rate of 0.5 Å s^−1^ for a 100 nm Ag finger.

### 4.3 Device Fabrication of Monolithic Perovskite/TOPCon Silicon Tandem Solar Cells

The fabrication of the silicon bottom cell followed the protocol outlined in the Supporting Information of our previous study [31, 48, 49]. Cleaned substrates for the perovskite top cells were UV–ozone treated for 15 min, then immediately moved into the N_2_ glovebox. The deposition procedures for the Me‐4PACz (GuaSCN) mixture, perovskite layer, C_60_, and BCP were identical to those used for single‐junction PSCs. A 1 nm Ag layer was deposited on top of the BCP. A transparent indium zinc oxide (IZO) electrode (30 Ω sq^−1^,90 nm) was subsequently deposited at room temperature via radio frequency (RF) magnetron sputtering at 80 W. Ag fingers (∼200 nm thick, 40−45 µm wide) were then thermally evaporated with a deposition rate of 0.5 Å s^−1^. Finally, a 100 nm MgF_x_ antireflection layer (99.9%, Plasmaterials) was thermally evaporated at the same rate.

### 4.4 Film Characterization

Using a Zetasizer Nano ZS (Malvern Instruments, UK), dynamic light scattering (DLS) measurements were conducted at 25 °C on SAM solutions dissolved in ethanol. Using a Nicolet 6700 spectrometer (Thermo Scientific, USA), Fourier transform infrared (FTIR) spectra were recorded, and an additional set of FTIR data was collected using a Jasco FT/IR‐6100 in the range of 4000−400 cm^−1^. Surface potentials were measured by Kelvin probe force microscopy (KPFM) using a Dimension 3100 (Veeco, USA). Ultraviolet photoelectron spectroscopy (UPS) measurements were performed using an Axis Ultra DLD system (Kratos, UK) under ultrahigh vacuum (∼2.0 × 10^−8^ Torr) with non‐monochromatic He I radiation at 21.22 eV. ^1^H nuclear magnetic resonance (^1^H‐NMR) spectra were using DMSO‐d_6_ (2.50 ppm) as the solvent, and measured on an AVANCE NEO 600 spectrometer (Bruker, Switzerland). X‐ray diffraction (XRD) and grazing‐incidence XRD (GIXRD) patterns were obtained using a D8 Advance DAVINCI (Bruker, Germany) with Cu Kα_1_ radiation (λ = 1.5418 Å). Depth‐resolved GIXRD was carried out using a Smart Lab five‐axis diffractometer (Rigaku, Japan) equipped with Cu Kα radiation (λ = 1.5405 Å), operated at 45 kV and 200 mA. The scan rate is 0.15°min^−1^, which was used to resolve residual stress information in the perovskite layers. The surface and cross‐sectional morphologies of the perovskite and tandem structures were analyzed using field‐emission scanning electron microscopy (FE‐SEM, Hitachi S‐4800, Japan). Surface roughness (RMS) values were obtained using an atomic force microscope (AFM, Dimension 3100, Veeco, USA). Photoluminescence quantum yield (PLQY) was measured in two configurations: (1) perovskite on glass; and (2) ITO/SAM/perovskite. A 532 nm continuous‐wave laser (Class 3b) served as the excitation source and was directed into an integrating sphere via optical fiber. The excitation intensity was calibrated to 1 sun by adjusting the laser power such that the perovskite film sized 2.5 × 2.5 cm^2^, tested under short‐circuit conditions, generated a photocurrent equivalent to the *J_SC_* measured under standard AM 1.5G illumination (20.9 mA cm^−2^ at 100 mW cm^−2^). Steady‐state and time‐resolved photoluminescence (SSPL and TRPL) were measured with an FLS920 fluorescence spectrometer (Edinburgh Instruments, UK).

### 4.5 Device Characterization

The *J−V* performance was evaluated using a Keithley 2400 source meter, under simulated solar illumination (AM 1.5G, 1 sun, 100 mW cm^−2^) provided by an Enlitech SS‐F5‐3A system. A shadow mask defining a 0.1 cm^2^ active area was employed during *J−V* characterization of the perovskite solar cells in ambient air. Measurements based on the space‐charge‐limited current (SCLC) method were conducted under identical conditions in the absence of light. EQE measurements were acquired using a QE‐R system (Enlitech, Taiwan). Light intensity at each wavelength was calibrated using certified single‐crystal Si and Ge reference cells. For spectral separation, the top perovskite cell was measured with a long‐pass filter (>800 nm), while a low‐pass filter (<800 nm) was used for the silicon bottom cell. A bias voltage of approximately 0.15 V was applied during EQE measurements to ensure short‐circuit conditions. EQE analyses were carried out in an ambient atmosphere without sample encapsulation. A Zahner 1240 A impedance analyzer was utilized to conduct EIS, C–F, and TPV measurements. Mott–Schottky measurements were carried out by recording impedance–potential responses under applied bias voltages ranging from −1.2 to 0.5 V.

### 4.6 Long‐Term Stability Test

The solar cell aging test system (91 PVK SOLAR) equipped with an LED simulating the AM 1.5G spectrum was used to perform maximum power point (MPP) tracking. *J−V* scans were recorded every 10 h to monitor the maximum power output (P_max_) during aging. For the monolithic perovskite/TOPCon silicon TSCs, the LED was switched out for a Xenon lamp, while other test conditions as described in our previous work. The operational stability of both single‐junction and TSCs was evaluated following the ISOS‐L‐1 protocol under continuous illumination in ambient air (25 °C−35 °C, 40%−60% relative humidity) without encapsulation. Furthermore, single‐junction devices were evaluated at 65 °C in a nitrogen atmosphere under continuous illumination following the ISOS‐L‐2 protocol.

## Author Contributions

H.M., Z.Y., X.L., M.X., X.Y., and J.Y. performed conceptualization. H.M. performed data curation. H.M., Z.Y., X.L., and X.Y. performed methodology. H.M., Z.Y., X.L., M.X., X.Y., and J.Y. performed an investigation. H.M., Z.Y., H.L., J.M., L.Z., J.W., Z.H., Y.Y., J.W., J.Y., X.Z., and M.Z. performed visualization. Z.Y., M.X., X.Y., and J.Y. acquired funding. Z.Y., X.L., Y.Z., M.X., X.Y., and J.Y. performed project administration. Z.Y., X.L., Y.Z., M.X., X.Y., and J.Y. performed supervision. H.M., Z.Y., X.L., Y.Z., M.X., X.Y., and J.Y. wrote the original draft. H.M., Z.Y., X.L., M.X., X.Y., and J.Y. wrote, reviewed, and edited the final manuscripts.

## Conflicts of Interest

The authors declare no conflicts of interest.

## Supporting information




**Supporting file**: advs74311‐sup‐0001‐SuppMat.docx.

## Data Availability

The data that support the findings of this study are available from the corresponding author upon reasonable request.
