# Supplementary material for: Cation–Anion Synergy Enables Uniform and Stable SAMs for High‐Efficiency Perovskite/TOPCon Tandem Solar Cells
Source: Adv Sci (Weinh). 2026 Feb 15;13(22):e20822. doi: 10.1002/advs.202520822 (PMC13088272; doi:10.1002/advs.202520822)
Supplement: Supplementary file 1 — Supporting file: advs74311‐sup‐0001‐SuppMat.docx. [file ADVS-13-e20822-s001.docx]

Supporting Information

**Cation–Anion Synergy Enables Uniform and Stable SAMs for High-Efficiency Perovskite/TOPCon Tandem Solar Cells**

*Haofan Ma^1,2^, Xin Li^2, *^, Huan Li^2^, Jianmin Guan^2^, Luyao Zheng^2^, Jun Wu^2^, Ziyu He^2^, Yunyun Yu^2^, Jungan Wang^4,5^, Jie Yang^4^, Xinyu Zhang^4,5^, Meili Zhang^2^,* *Yuheng Zeng^2^, Menglei Xu^4,5, *^, Zhiqin Ying^2, *^, Xi Yang^2,3^, Jichun Ye^2, 3, *^*


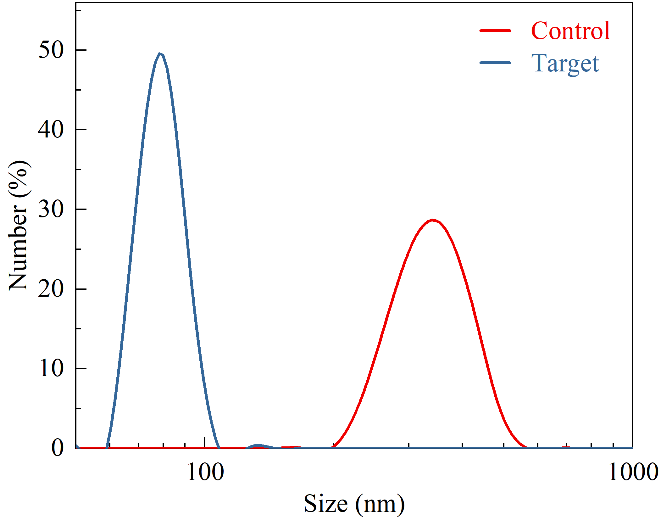


Figure S1. Particle size distribution in solutions of Me-4PACz and Me-4PACz (GuaSCN) as determined by DLS.


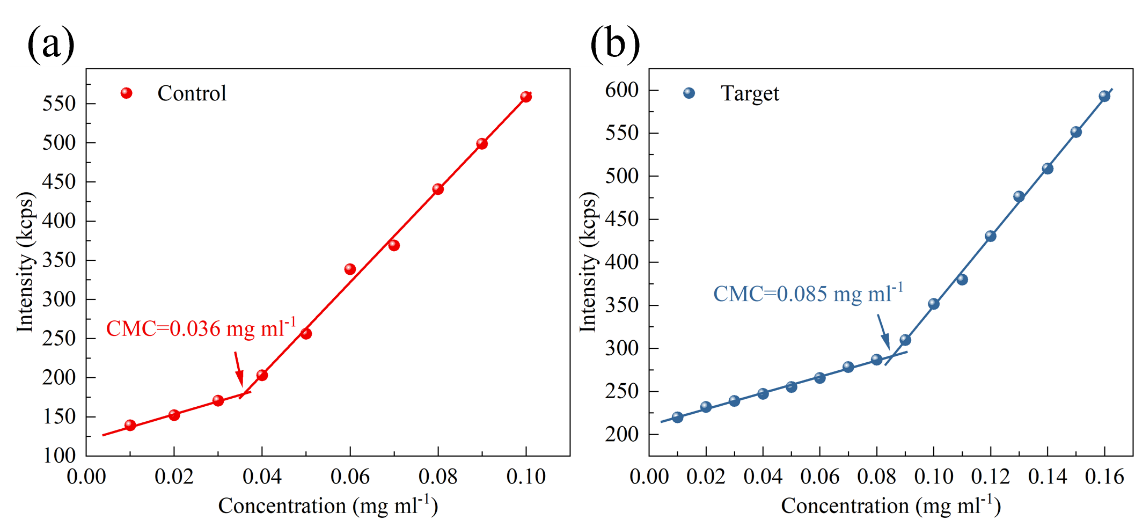


Figure S2. Light scattering intensity at different concentrations of (a)Me-4PACz; (b) Me-4PACz with GuaSCN in ethanol.


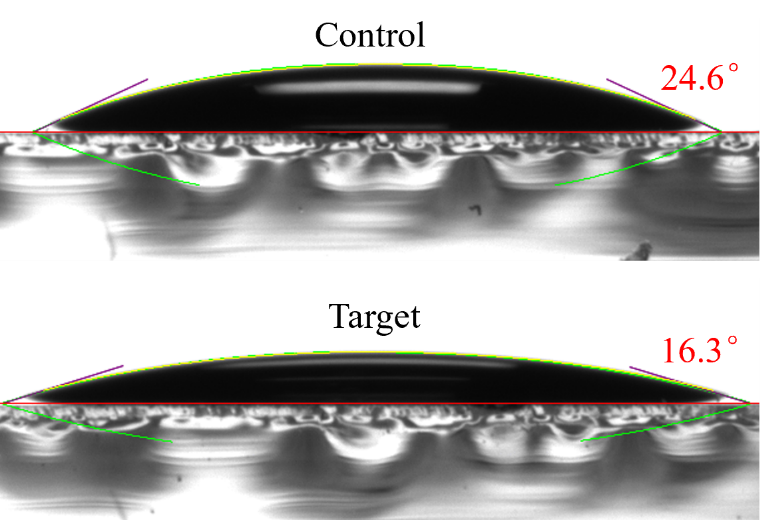


Figure S3. Contact angles of Me-4PACz /EtOH and Me-4PACz (GuaSCN)/EtOH SAM solutions on ITO surface.


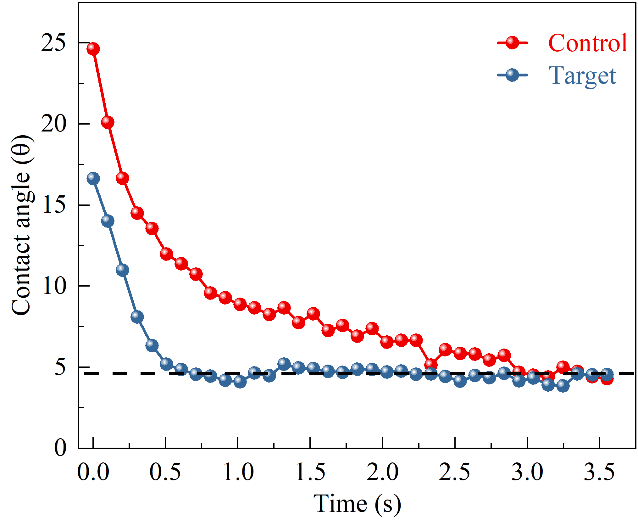


Figure S4. The change of contact angle of Me-4PACz solution on ITO substrate over time, with and without GuaSCN.


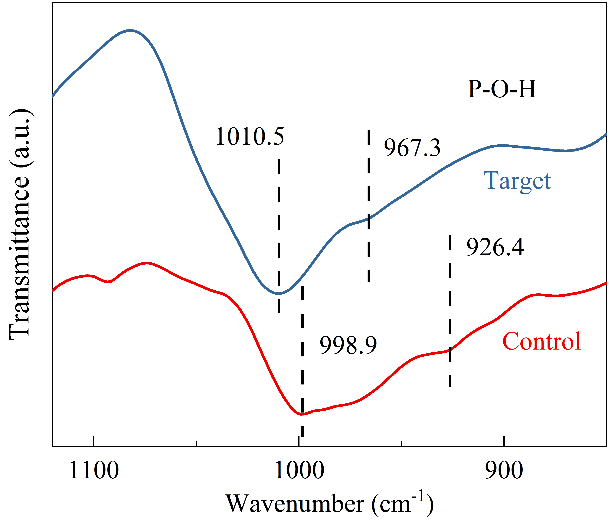


Figure S5. FTIR-ATR of Me-4PACz /EtOH and Me-4PACz (GuaSCN)/EtOH solutions.


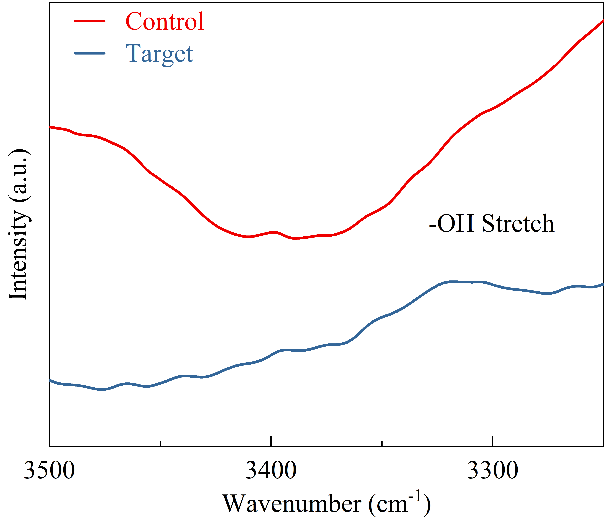


Figure S6. FTIR-ATR of Me-4PACz /EtOH and Me-4PACz (GuaSCN)/EtOH solutions.


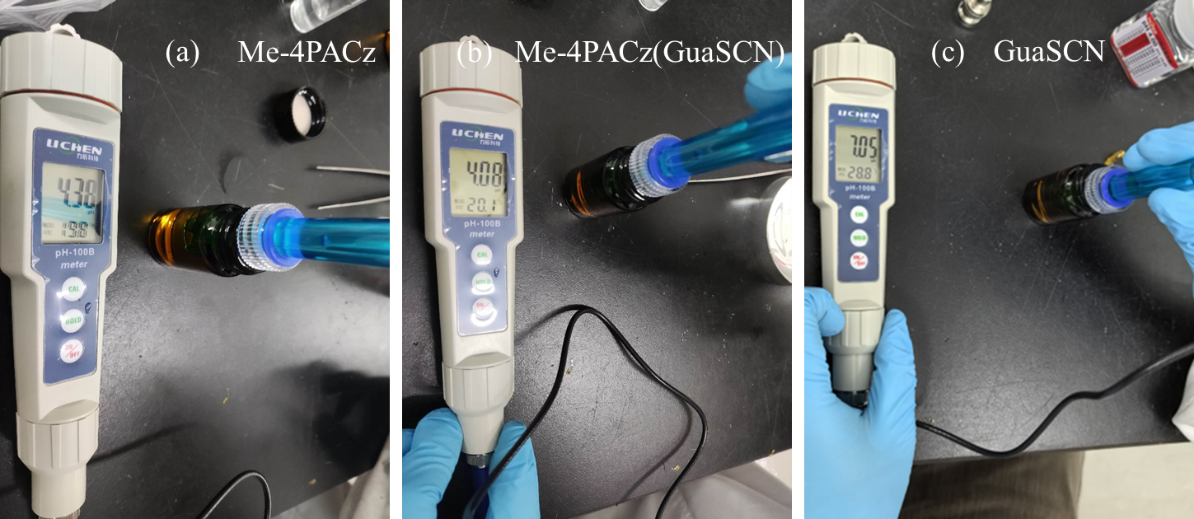


Figure S7. pH values of (a) Me-4PACz, (b) Me-4PACz with GuaSCN, and (c) GuaSCN in ethanol.


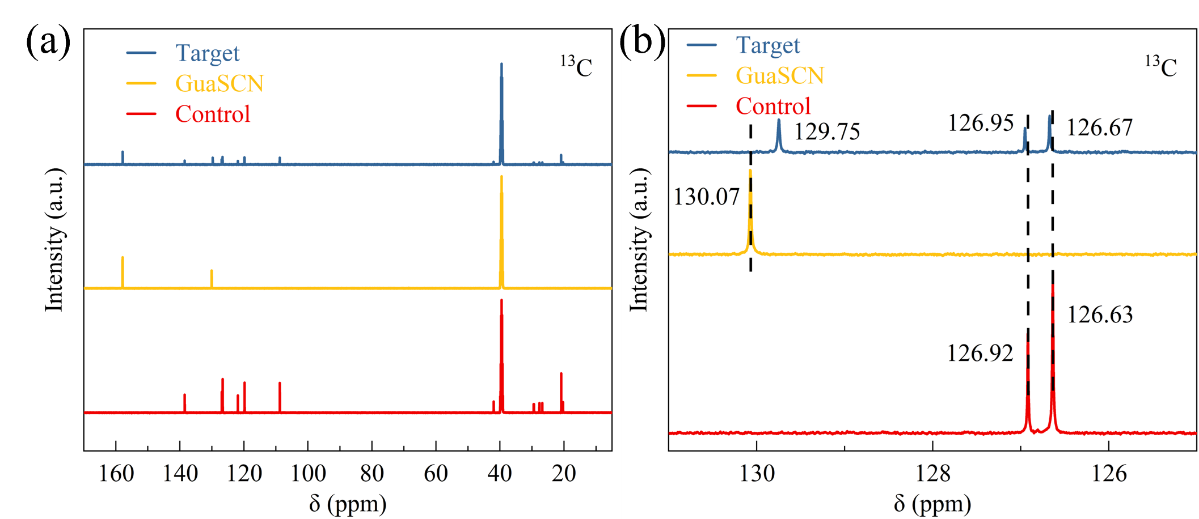


Figure S8. (a)^13^C-NMR spectra of Me-4PACz, GuaSCN and Me-4PACz + GuaSCN hybrid and (b) the corresponding local enlarged spectra.


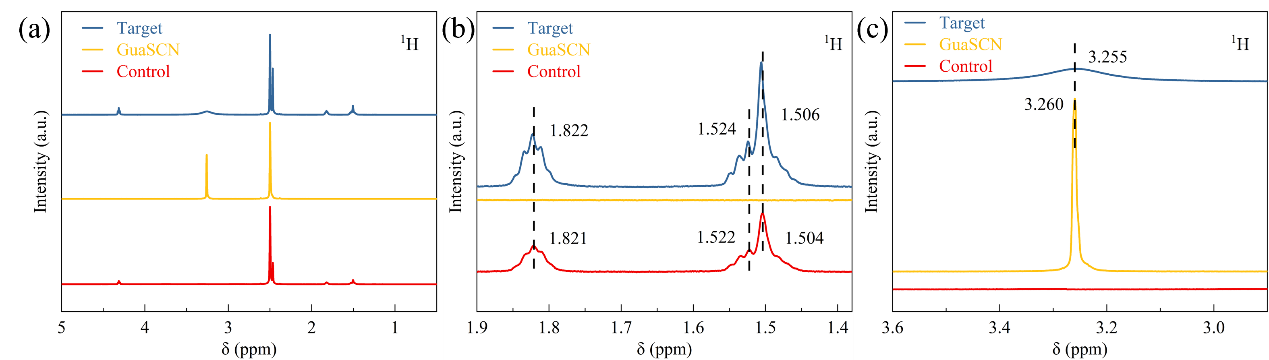


Figure S9. (a)^1^H-NMR spectra of Me-4PACz, GuaSCN and Me-4PACz + GuaSCN hybrid and (b, c) the corresponding local enlarged spectra.


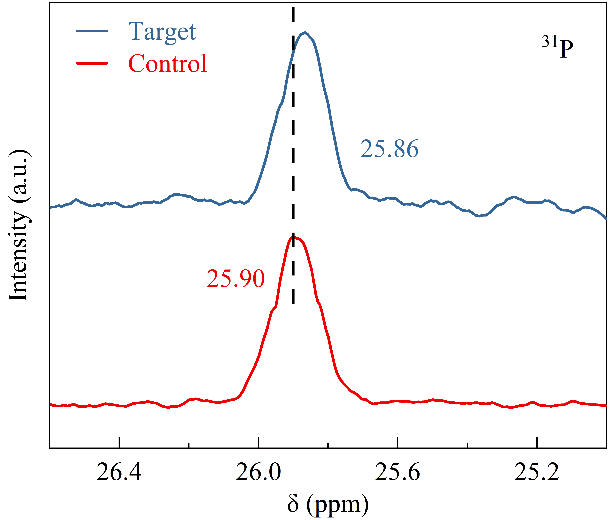


Figure S10. ^31^P-NMR spectra of Me-4PACz and Me-4PACz + GuaSCN hybrid.


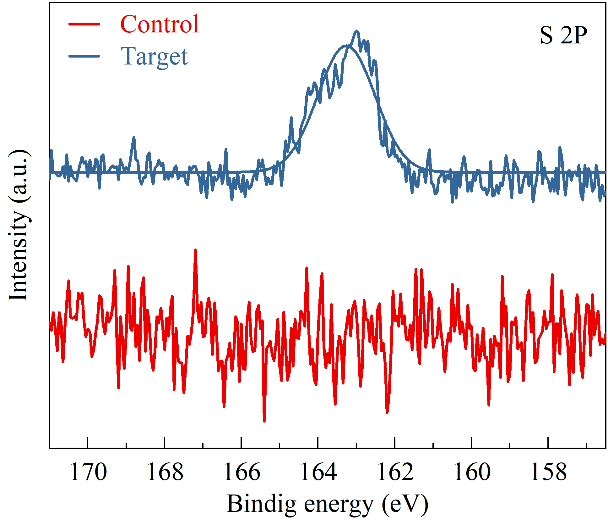


Figure S11. XPS spectra of S 2p for the ITO/Me-4PACz and ITO/Me-4PACz (GuaSCN) films.


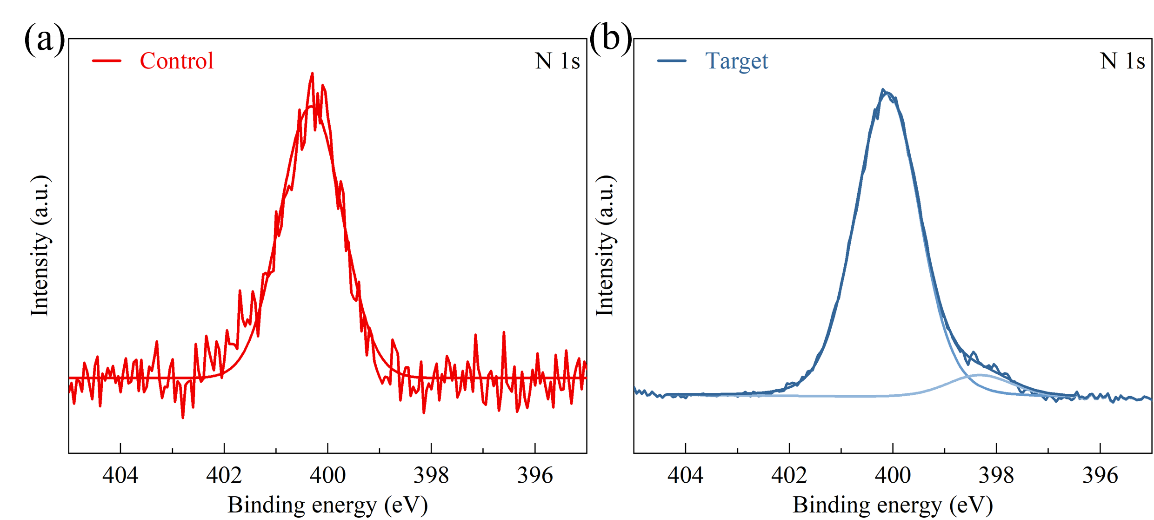


Figure S12. XPS spectra of N 1S for (a)ITO/Me-4PACz; (b) ITO/Me-4PACz (GuaSCN) films.


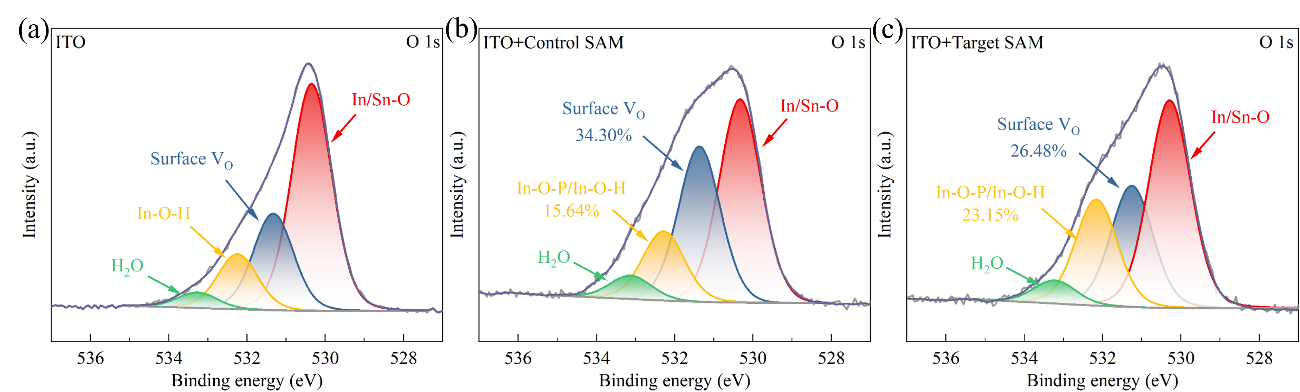


Figure S13. XPS spectra of O 1S for the ITO substrate.


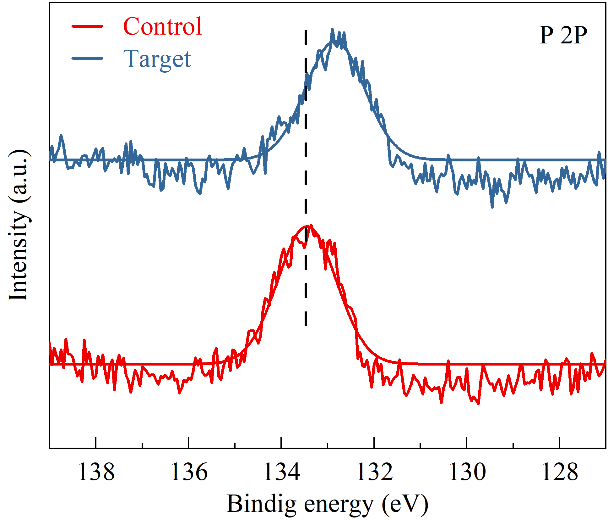


Figure S14. XPS spectra of P 2P for the ITO/Me-4PACz and ITO/Me-4PACz (GuaSCN) films.


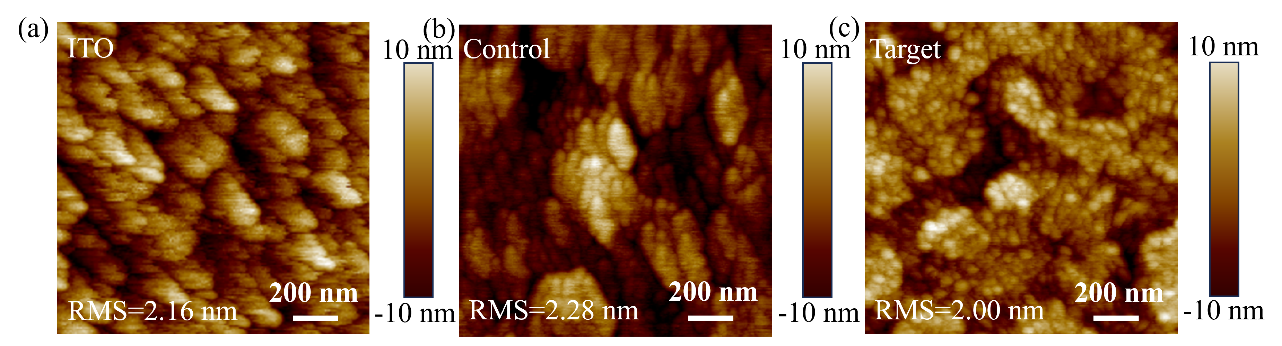


Figure S15. AFM images of (a) ITO; (b) ITO/Me-4PACz; (c) ITO/Me-4PACz (GuaSCN) films.


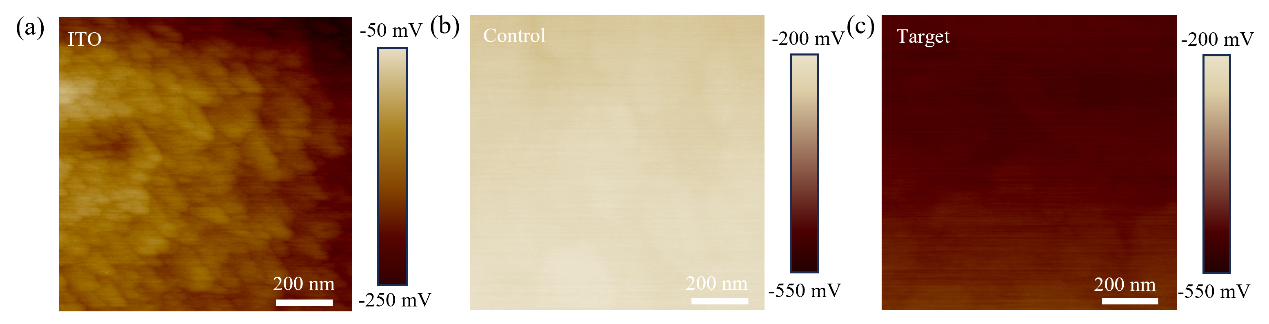


Figure S16. KPFM images of (a) ITO; (b) ITO/Me-4PACz; (c) ITO/Me-4PACz (GuaSCN) films.


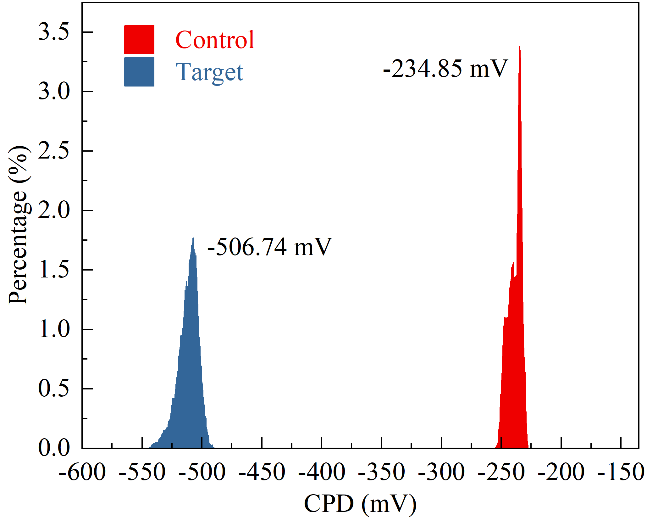


Figure S17. The CPD distribution histogram of the Me-4PACz and Me-4PACz (GuaSCN) films on ITO surface.


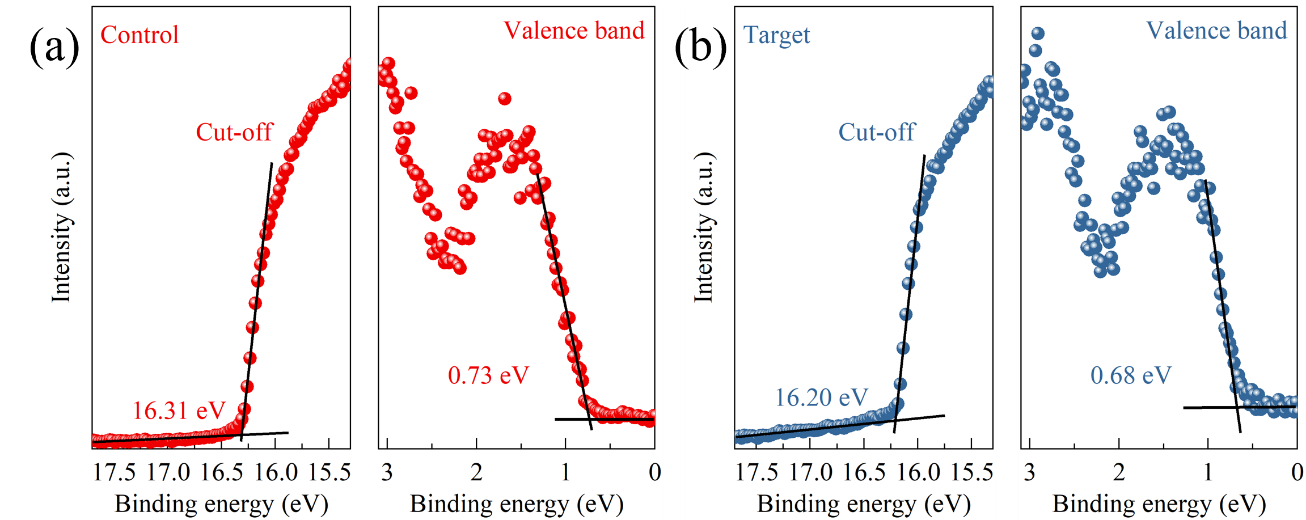


Figure S18. UPS cut-off edge and valence band spectra of (a) control; (b) Target films deposited on ITO substrate, respectively.


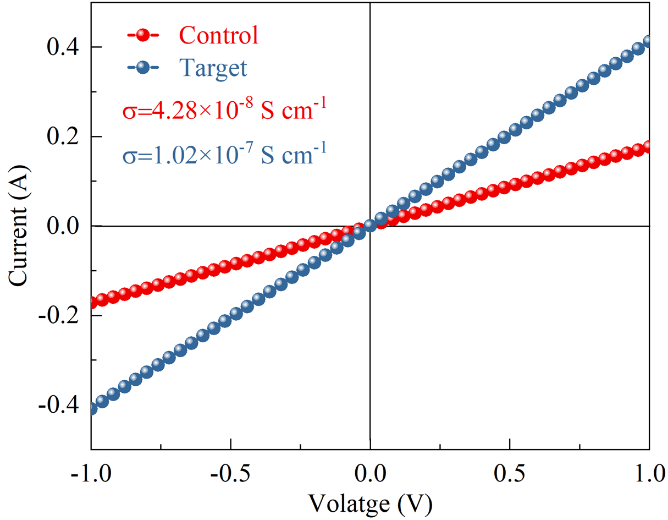


Figure S19. Conductivity of Me-4PACz and GuaSCN treated Me-4PACz films.


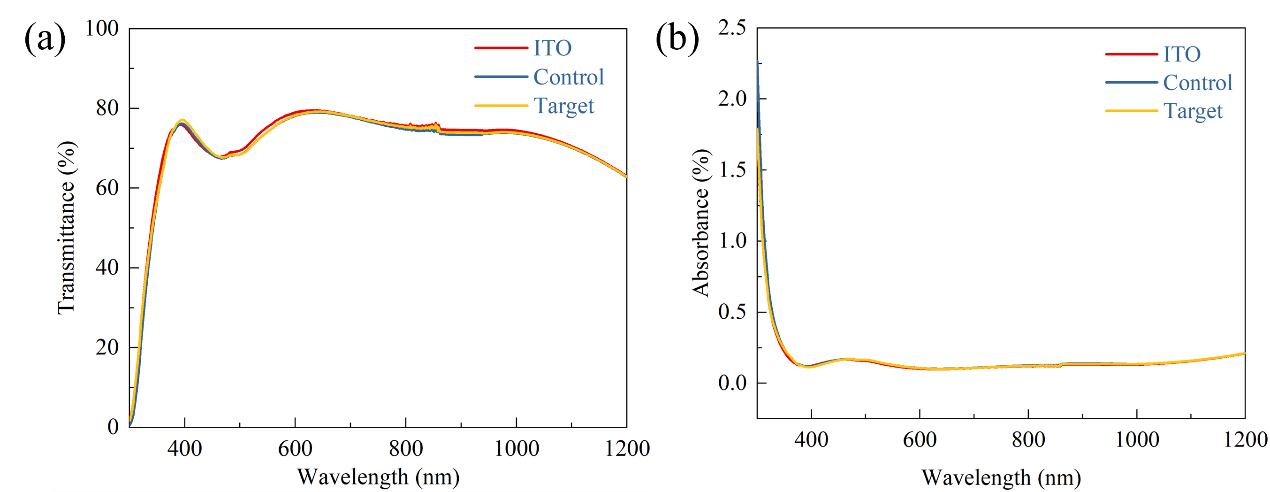


Figure S20. UV–Vis spectra of ITO, Me-4PACz, and Me-4PACz(GuaSCN) films: (a) transmittance and (b) absorbance.


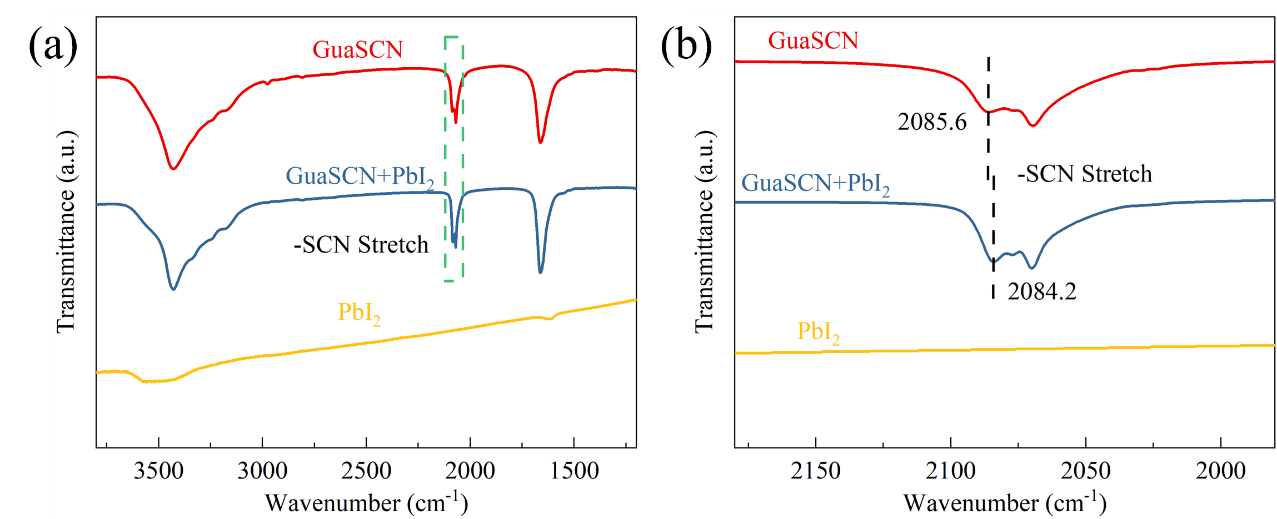


Figure S21. (a) FTIR spectra of the GuaSCN, GuaSCN+PbI_2_ and PbI_2_; (b) the corresponding local enlarged spectra.


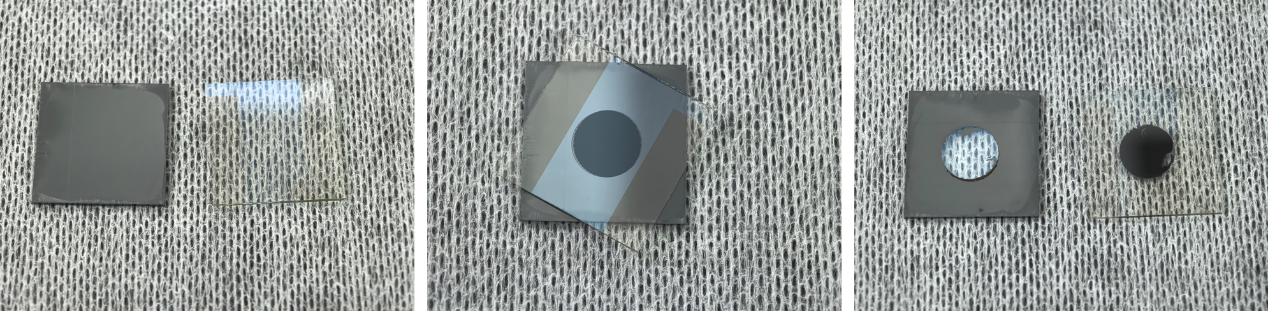


Figure S22. Exfoliating process of perovskite film


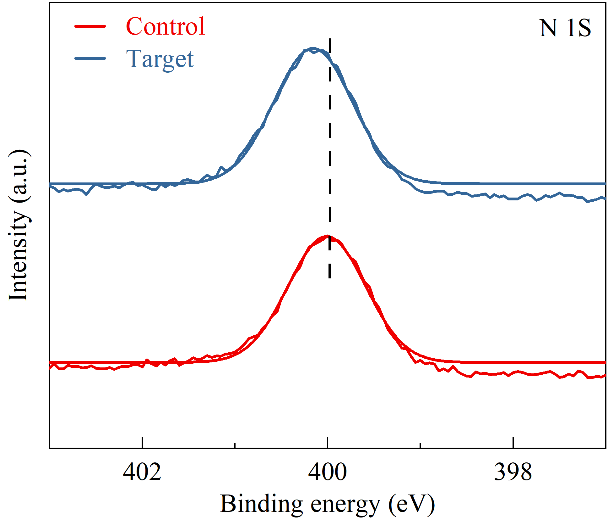


Figure S23. XPS spectra of N 1S for perovskite films exposed the buried surface without and with the treatment of GuaSCN.


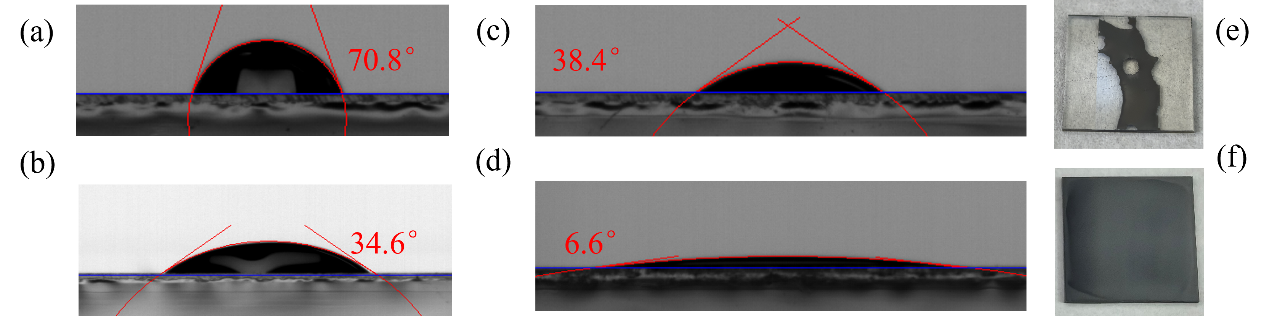


Figure S24. (a) and (b) Contact angles for deionized water on the control and target film; (c) and (d) Contact angles for perovskite precursor solution on the control and target film;(e) and (f) Perovskite film coating area on the control and target films.


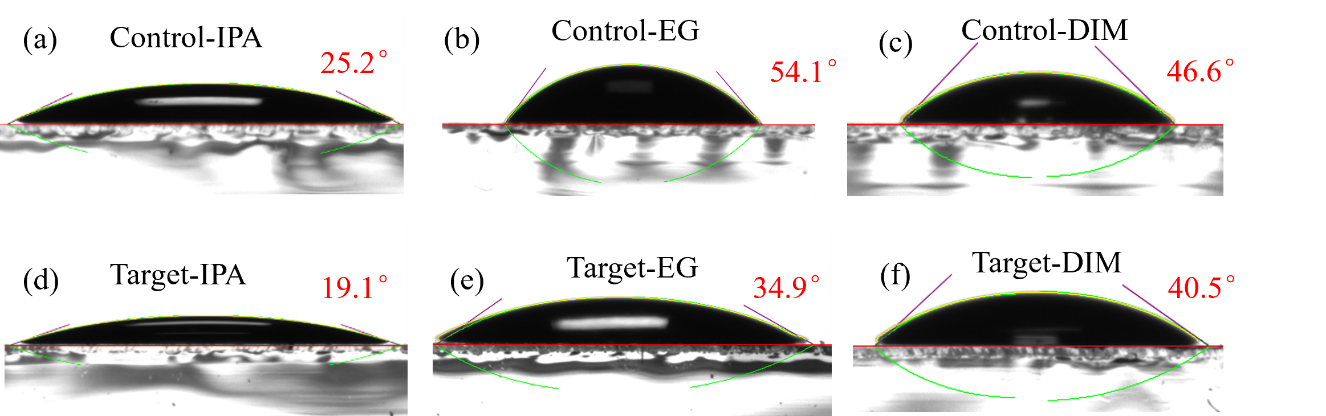


Figure S25. Contact angles on the control film by (a) Isopropanol; (b) Ethylene glycol; (c) Diiodomethane; Contact angles on the target film by (d) Isopropanol; (e) Ethylene glycol; (f) Diiodomethane.


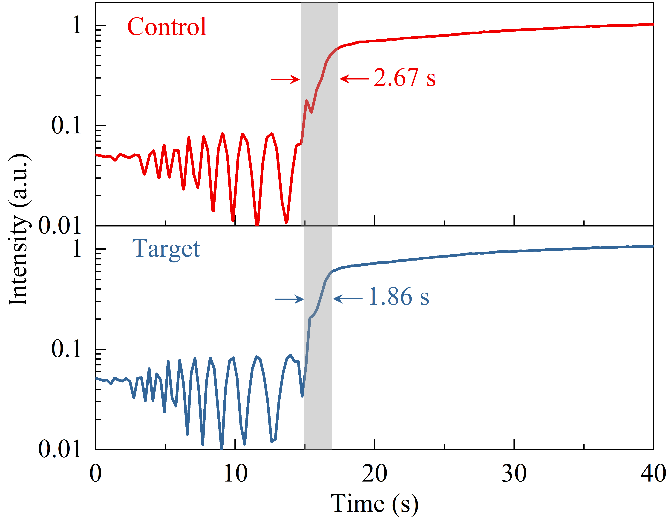


Figure S26. Time-dependent absorption evolution at 490 nm for the control and target perovskite precursor films.


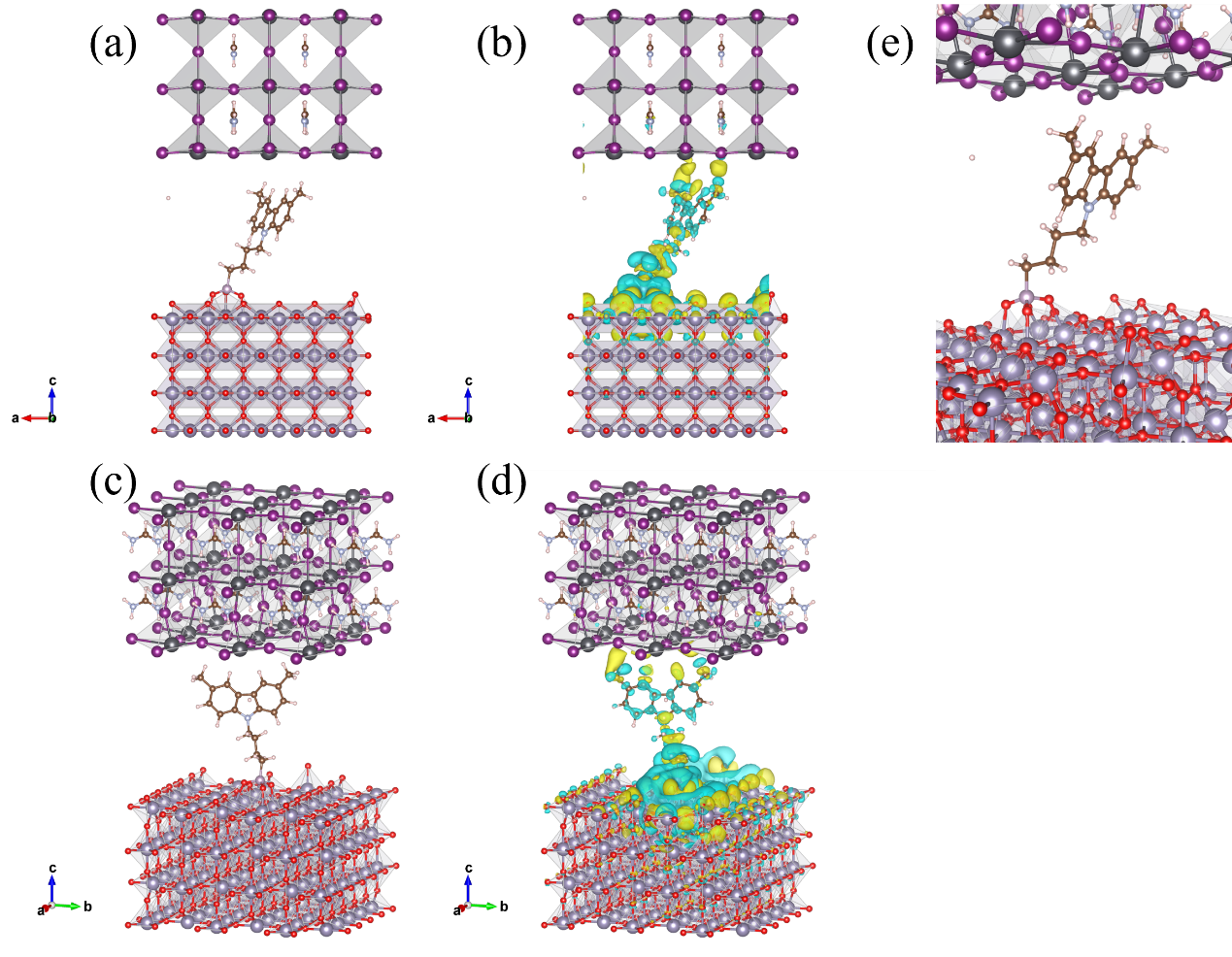


Figure S27. Different views and charge distribution of the ITO/Me-4PACz/PVK heterojunction structure obtained from DFT simulations.


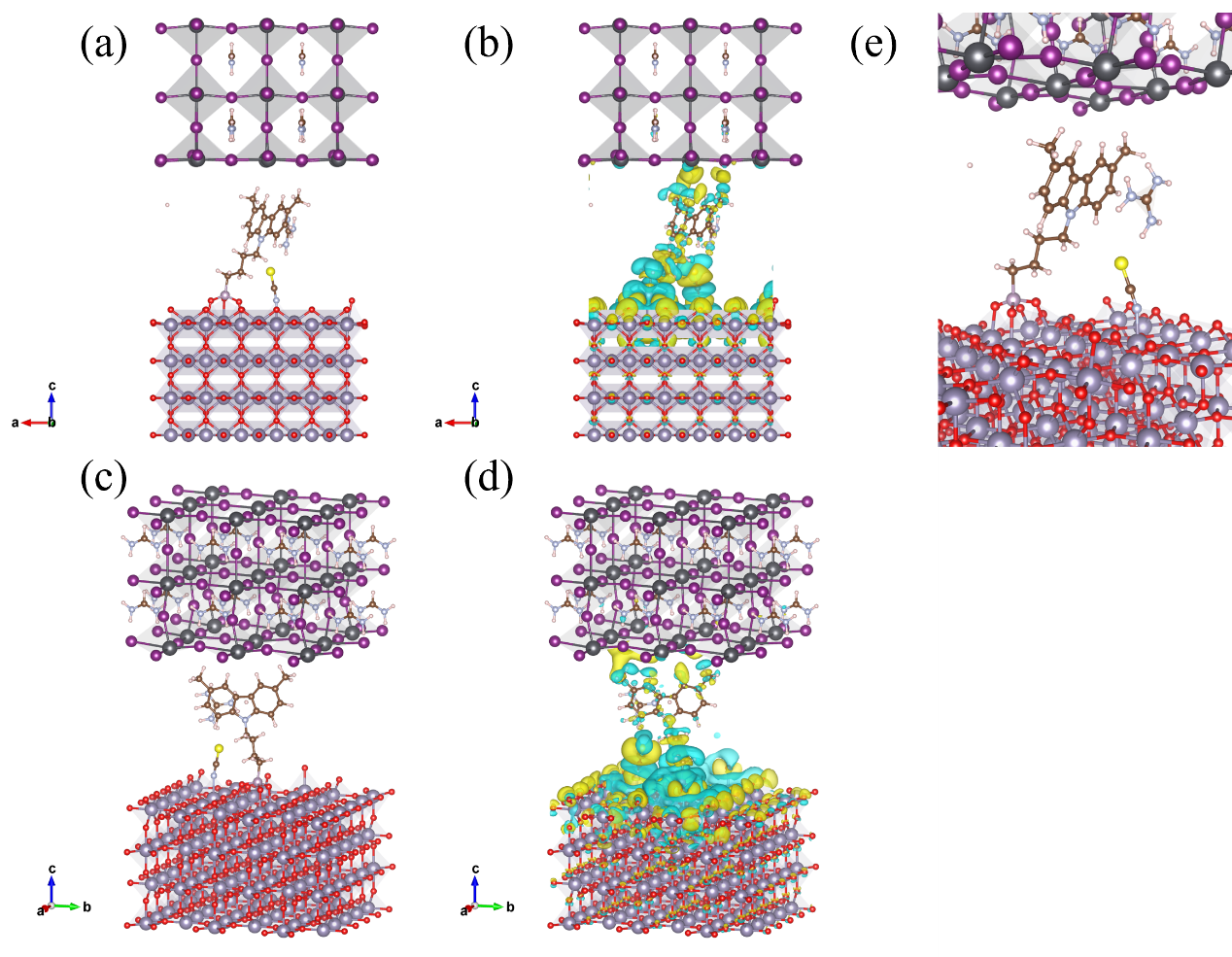


Figure S28. Different views and charge distribution of the ITO/ Me-4PACz (GuaSCN)/PVK heterojunction structure obtained from DFT simulations.


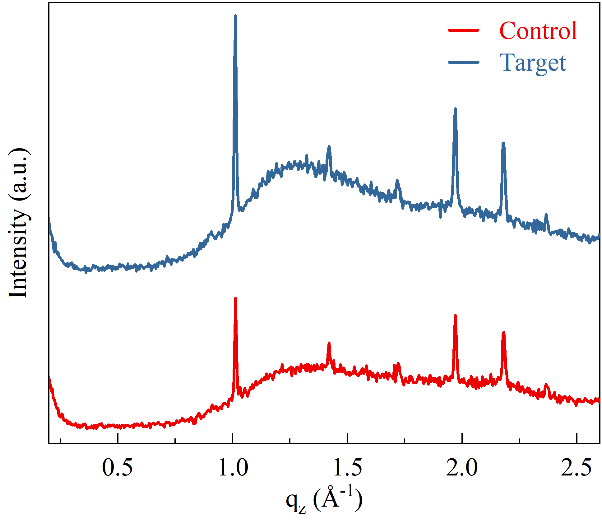


Figure S29. q_z_ GIWAXS patterns of the control and target perovskite films (exposed the buried surface) extracted from the corresponding 2D GIWAXS patterns.


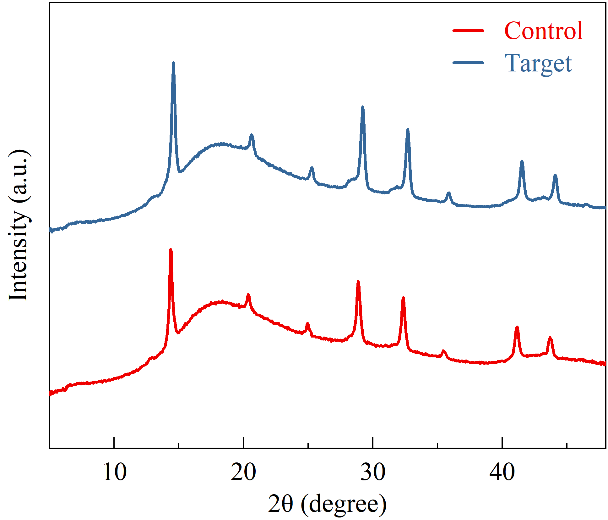


Figure S30. 2θ profiles extracted from GIWAXS measurements of perovskite films without and with GuaSCN modification.


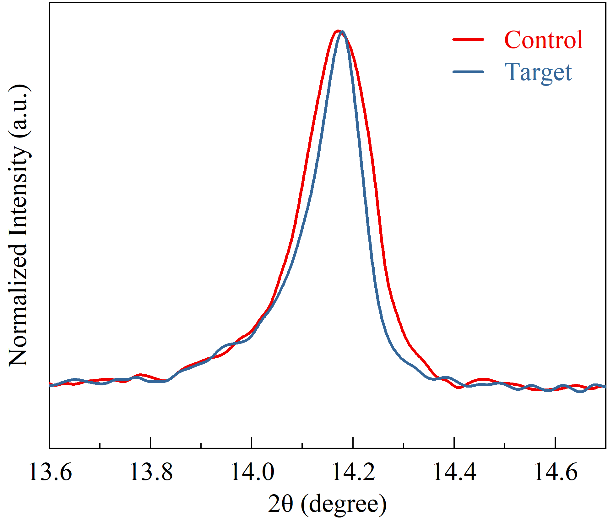


Figure S31. The full width at half maximum of the (100) plane of the perovskite film.

Figure S32. Top-SEM images of perovskite deposited on (a) control and (b) target films, and embedding graphs are grain size distribution maps.


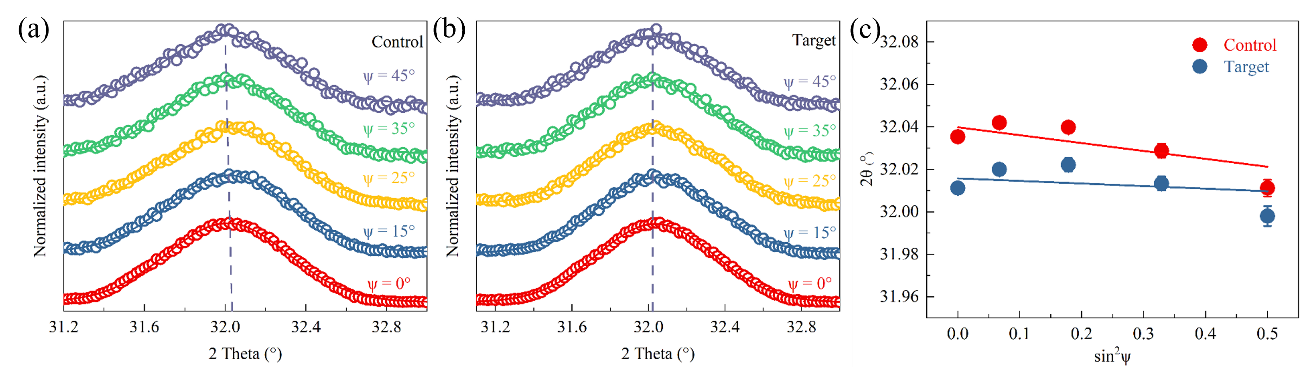


Figure S33. GIXRD patterns with different tilt angles for (a) control, (b) target perovskite films. (c) Linear fit of 2𝜃-sin2ψ for control and target perovskite films.


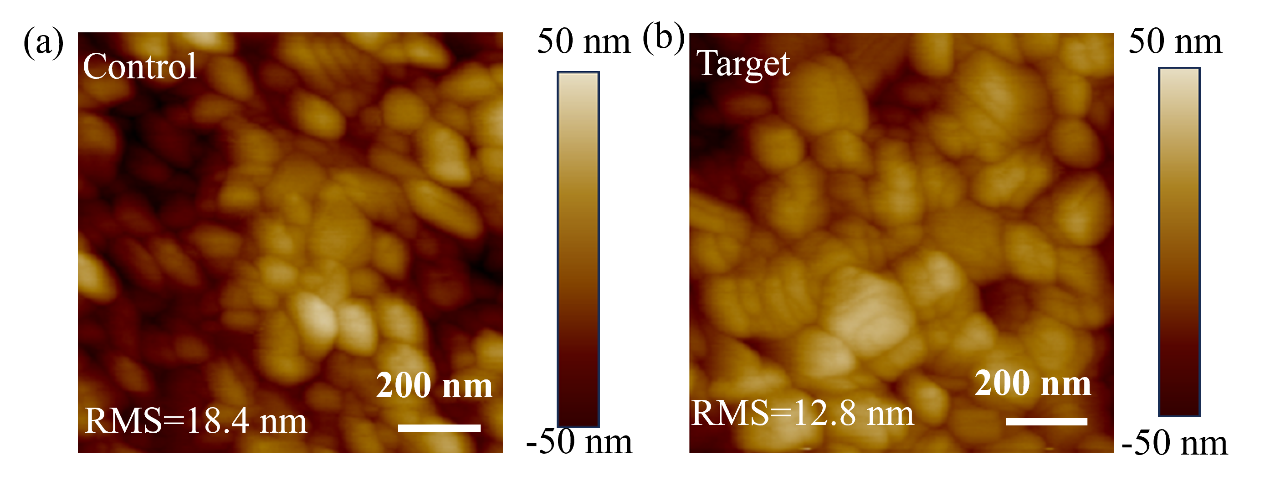


Figure S34. AFM images of (a) ITO/control SAM/PVK and (b) ITO/target SAM/PVK films.


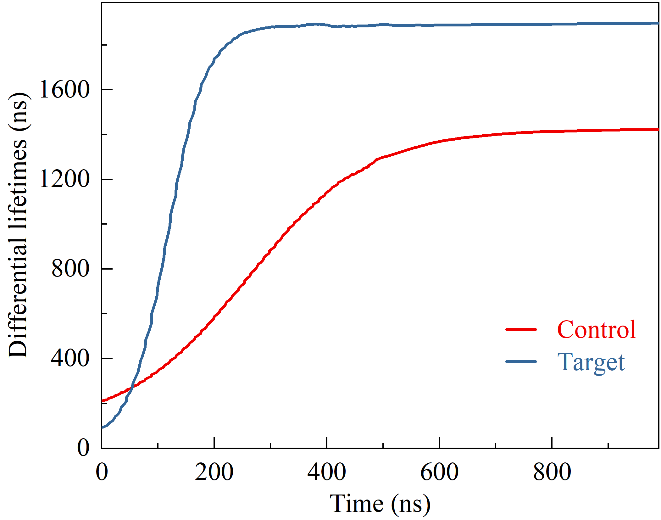


Figure S35. The differential lifetime of perovskite films deposited on control and target films from ITO side.


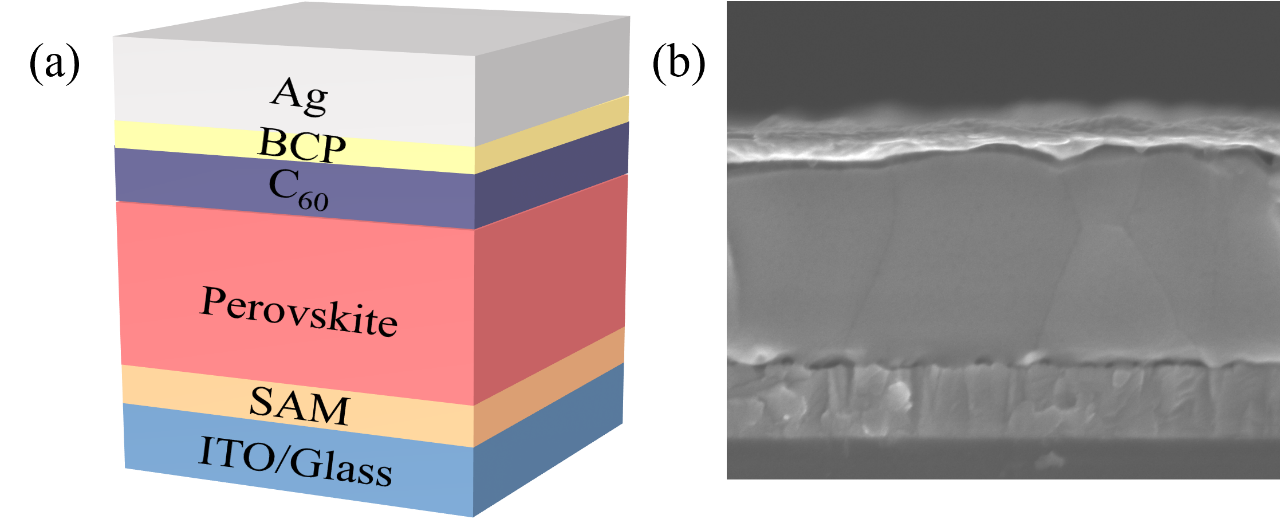


Figure S36. (a) Principle and (b) cross-sectional SEM image of single junction perovskite solar cells.


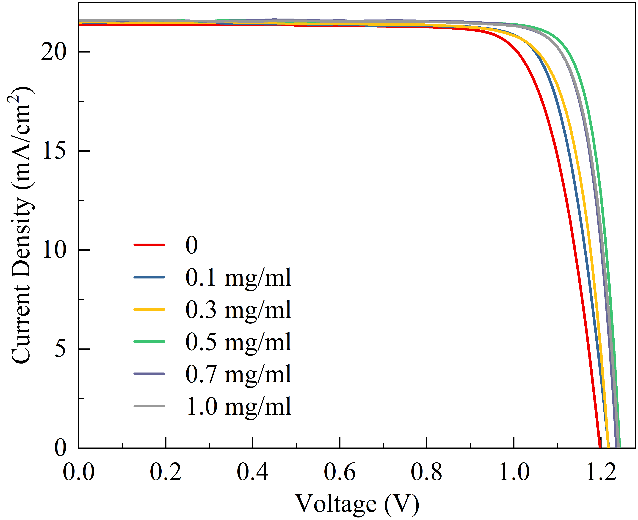


Figure S37. *J-V* curves of devices with different GuaSCN contents.


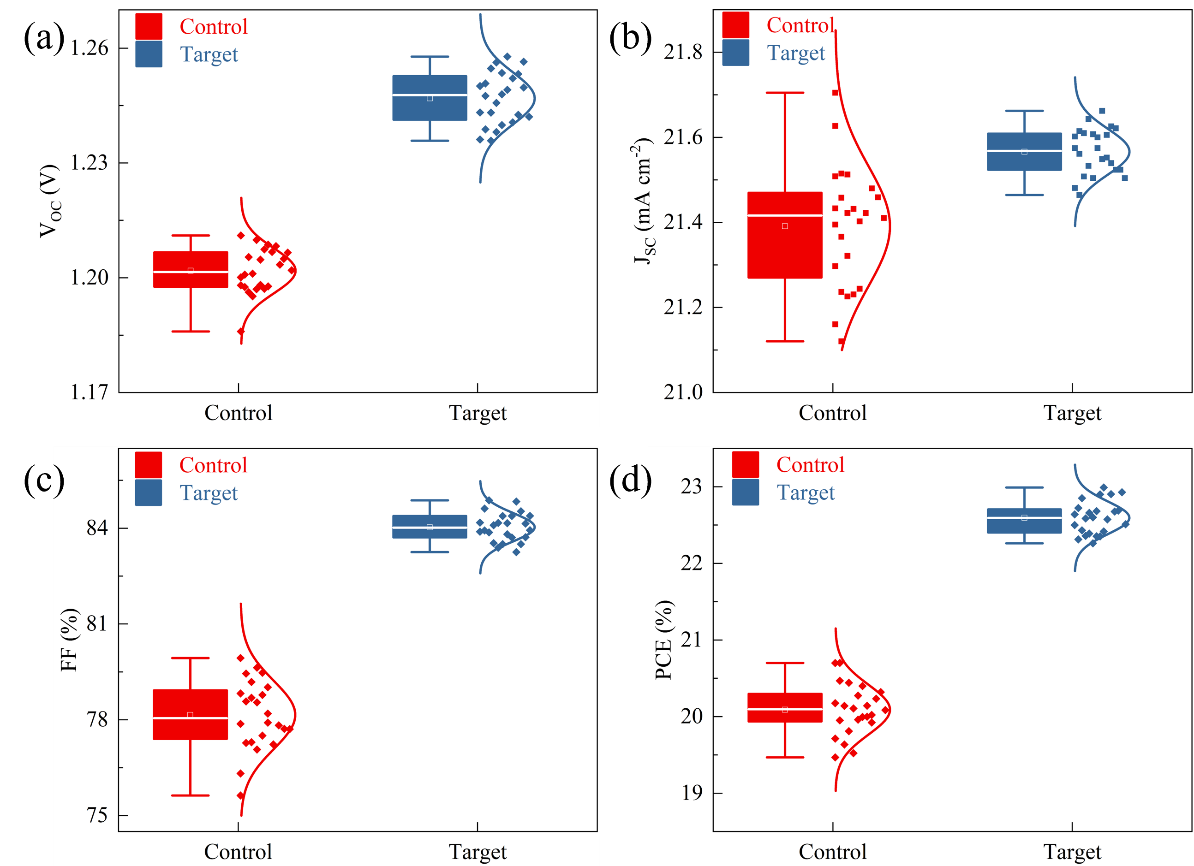


Figure S38. Statistical distributions of photovoltaic parameters for control and target devices. (a) Open-circuit voltage (*V*_OC_); (b) Short-circuit current density (*J*_SC_); (c) Fill factor (FF); (d) Power conversion efficiency (PCE).


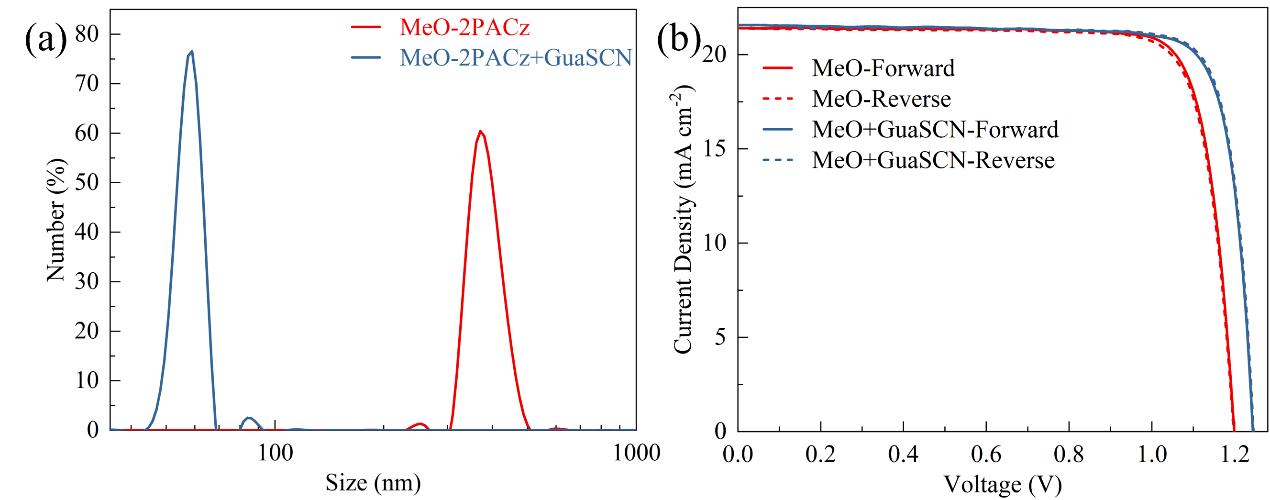


Figure S39. (a) Particle size distribution in solutions of MeO-2PACz and MeO-2PACz (GuaSCN) as determined by DLS; (b) *J−V* curves of the champion MeO-2PACz and MeO-2PACz (GuaSCN) based single-junction PSCs.


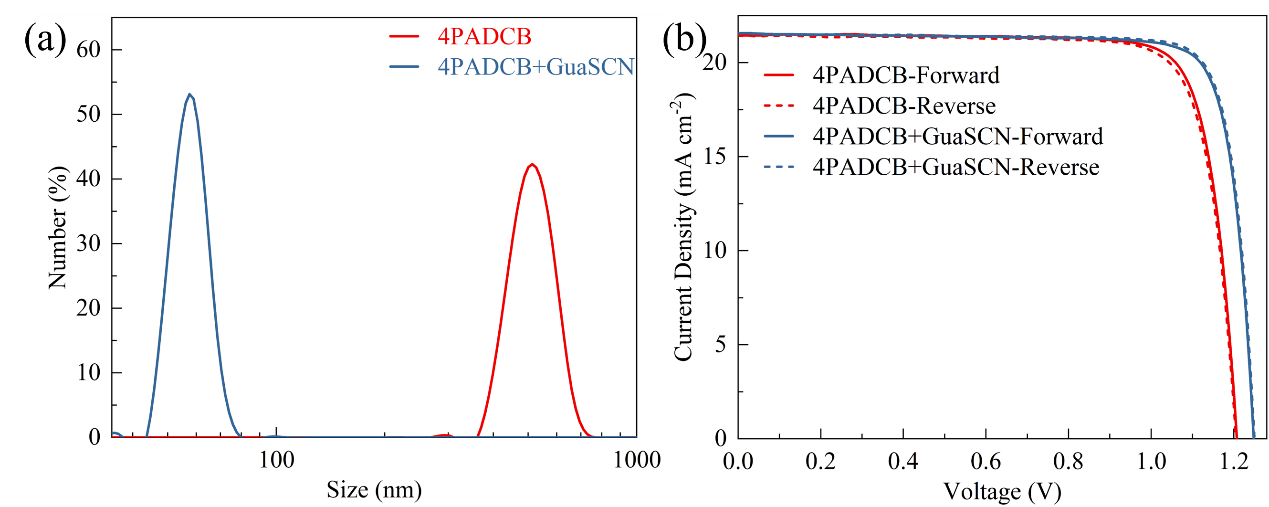


Figure S40. (a) Particle size distribution in solutions of 4PADCB and 4PADCB (GuaSCN) as determined by DLS; (b) *J−V* curves of the champion 4PADCB and 4PADCB (GuaSCN) based single-junction PSCs.


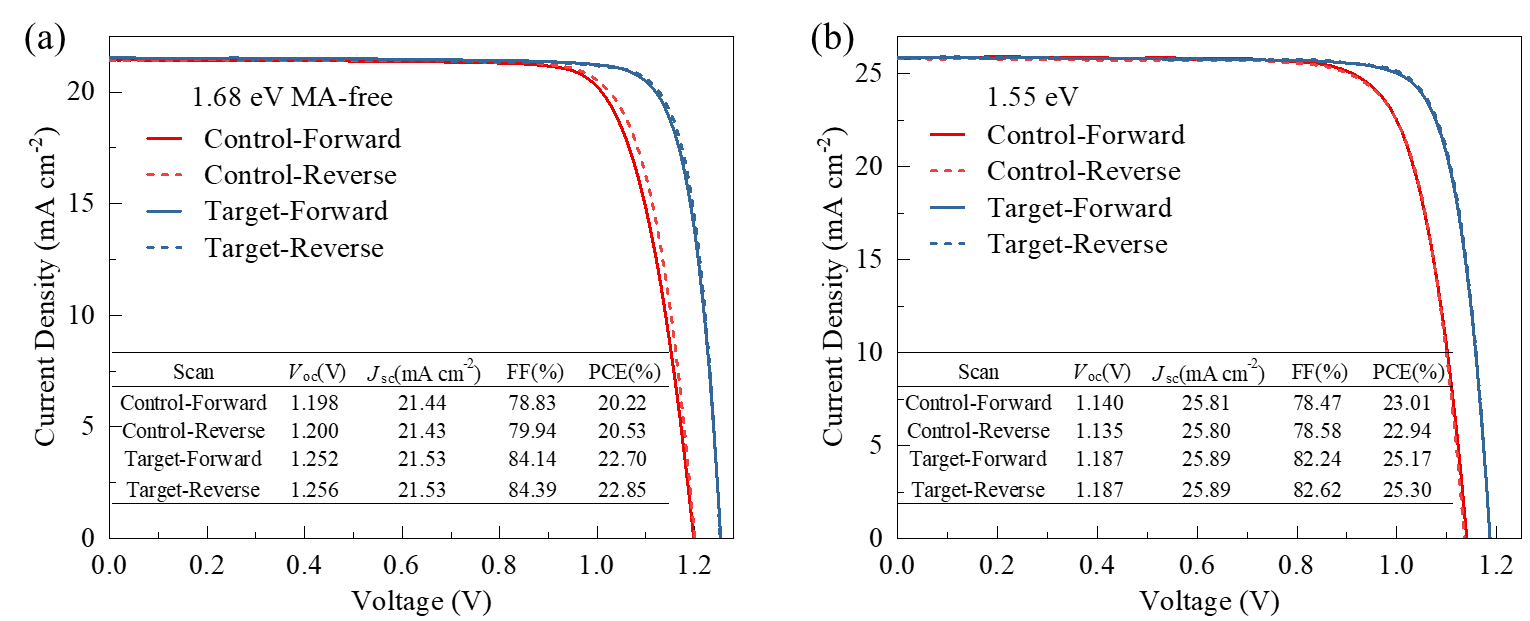


Figure S41. *J‒V* curves for (a) 1.68 eV MA-free and (b) 1.55 eV wide-bandgap perovskite solar cells.


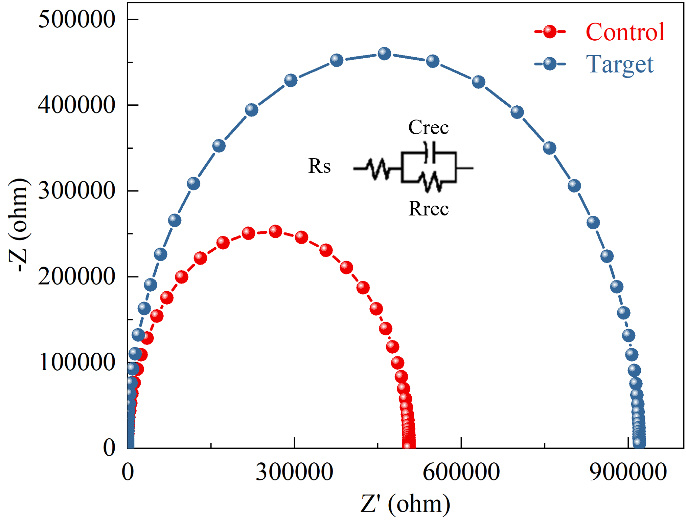


Figure S42. The electrochemical impedance spectroscopy (EIS) results of control and target devices. The inset shows the equivalent circuit diagram.


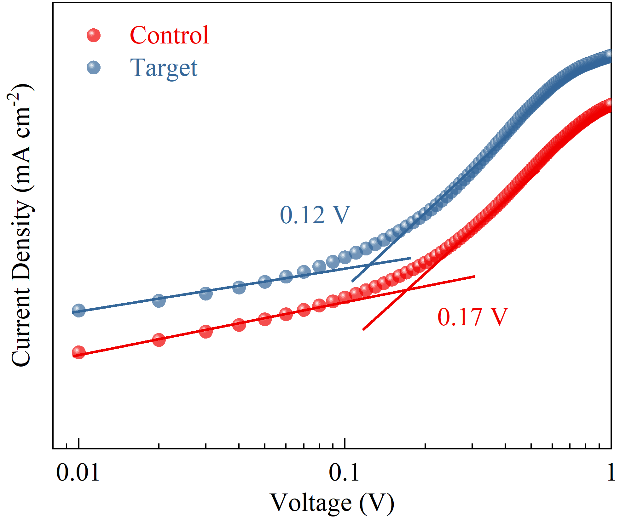


Figure S43. SCLC measurements for hole-only devices.


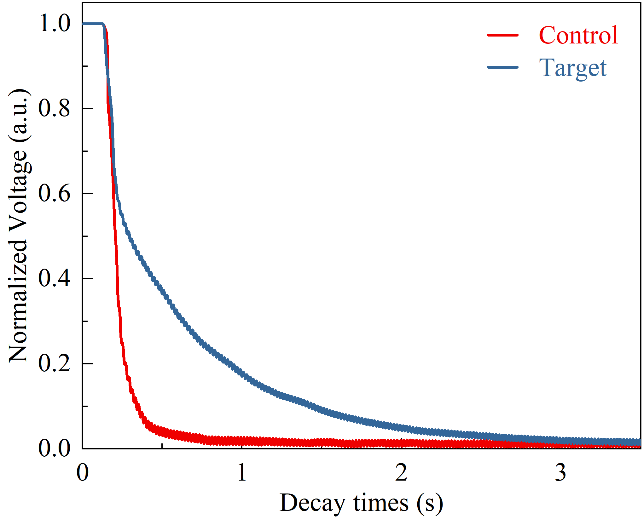


Figure S44. Transient photovoltage (TPV) decay curves of control and target devices.


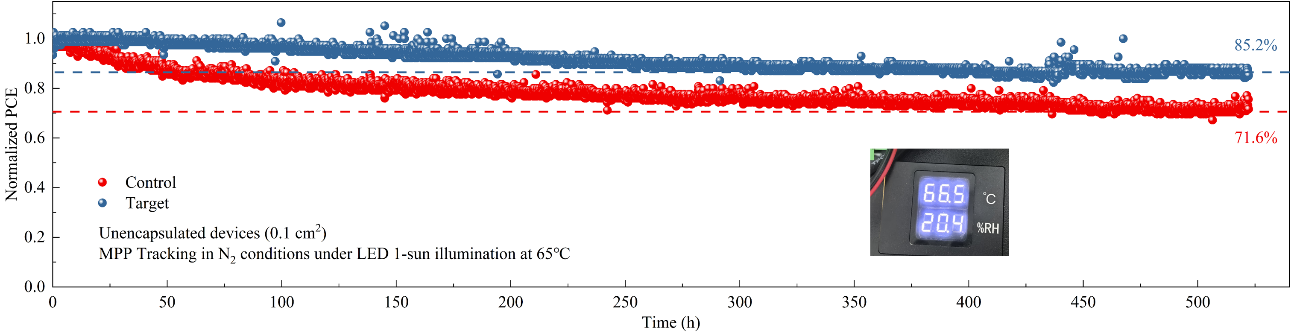
 Figure S45. Maximum power point tracking of encapsulated devices measured under continuous AM 1.5G illumination of 100 mW cm^-2^ at 65 ℃ and RH 20%.


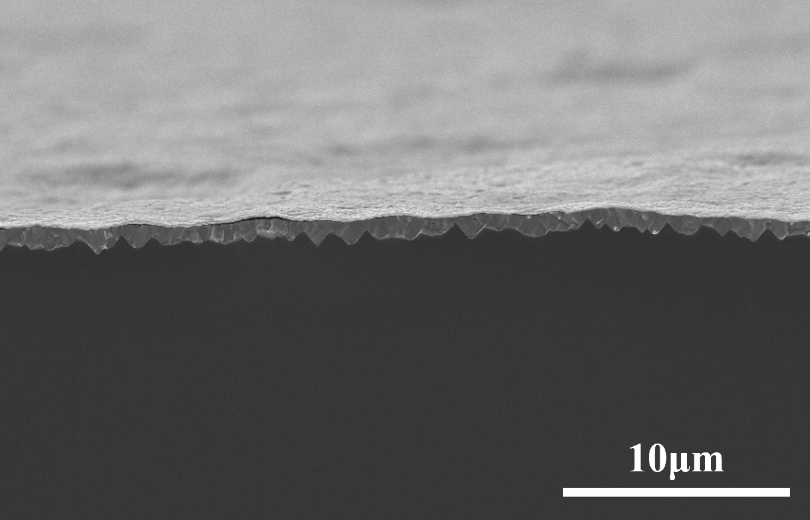


Figure S46. Cross-section SEM image of the perovskite/TOPCon silicon tandem solar cell in a broader area.


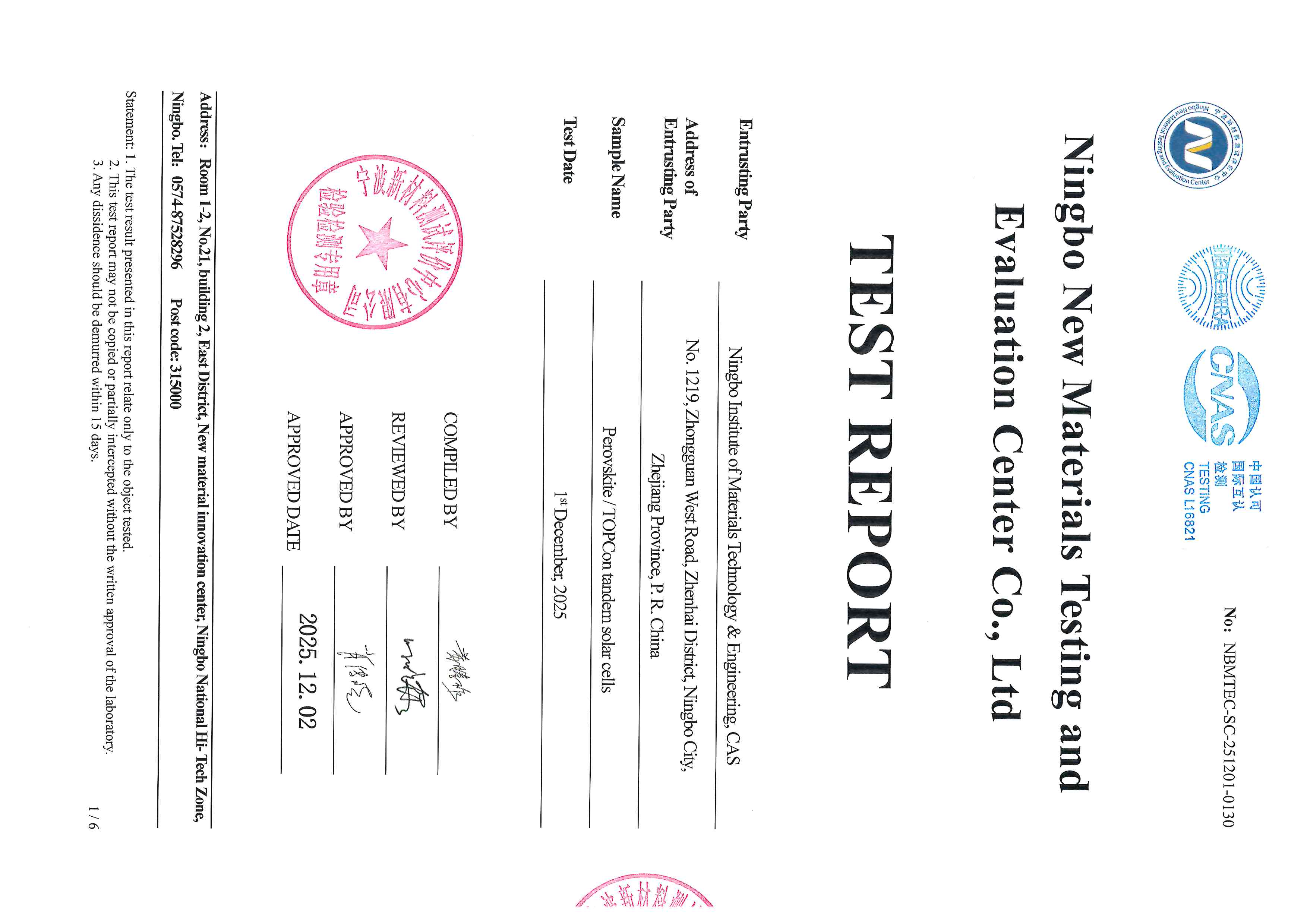


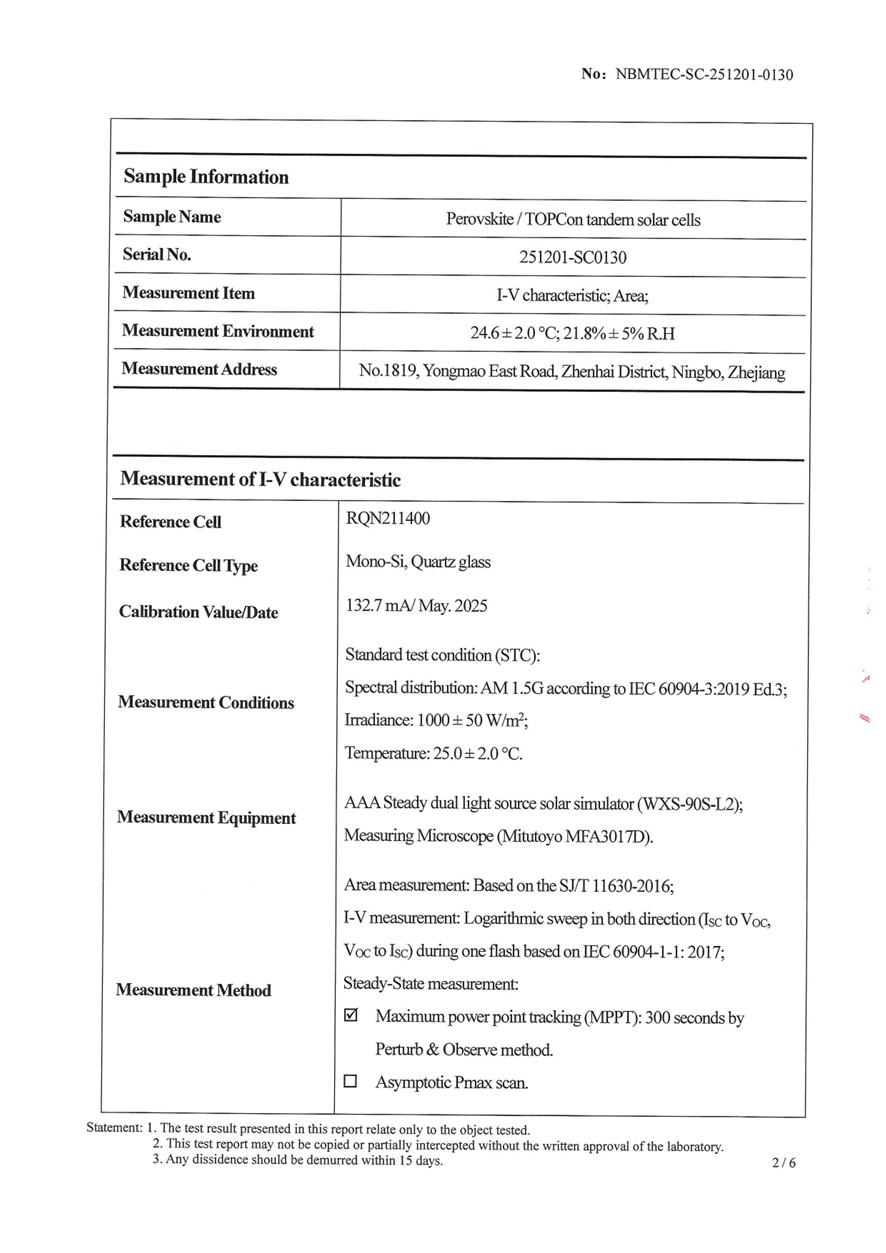

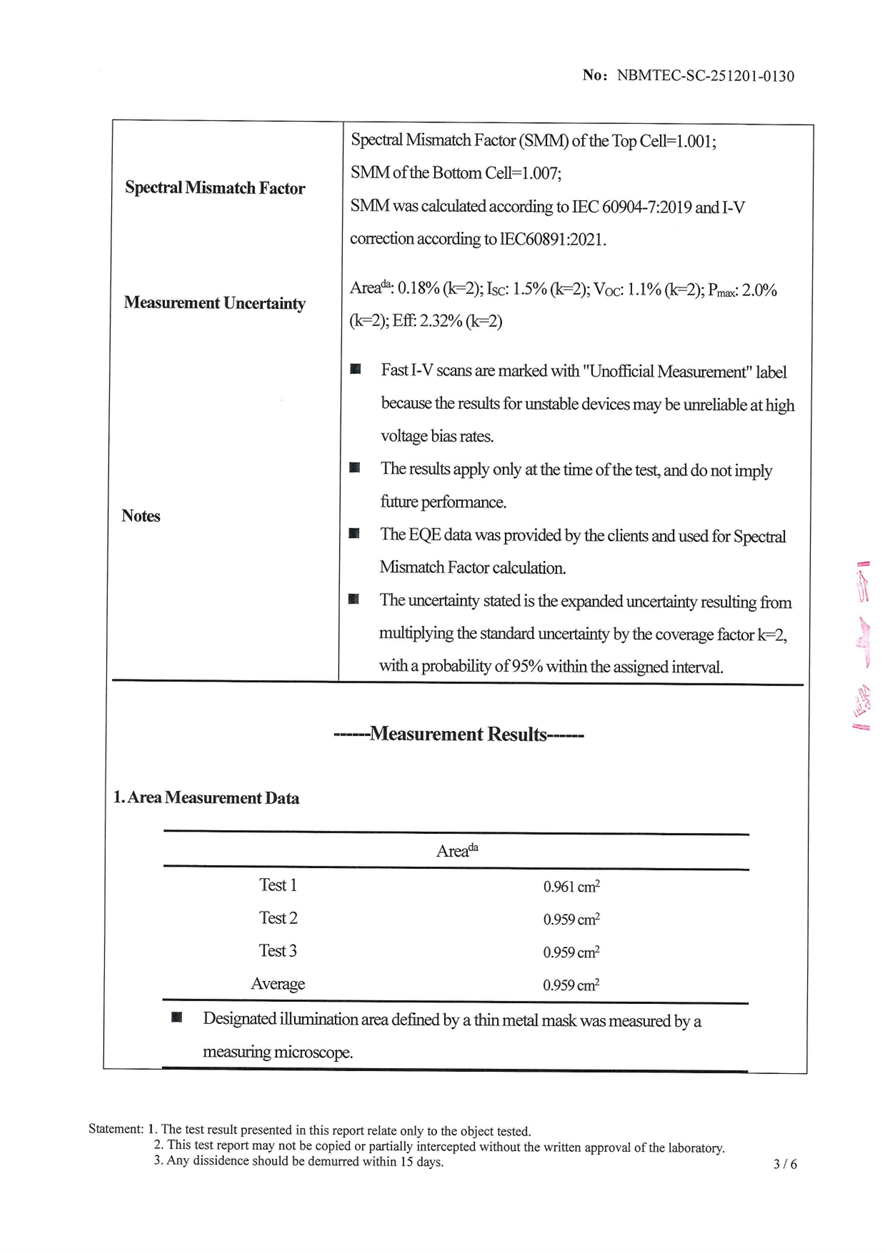

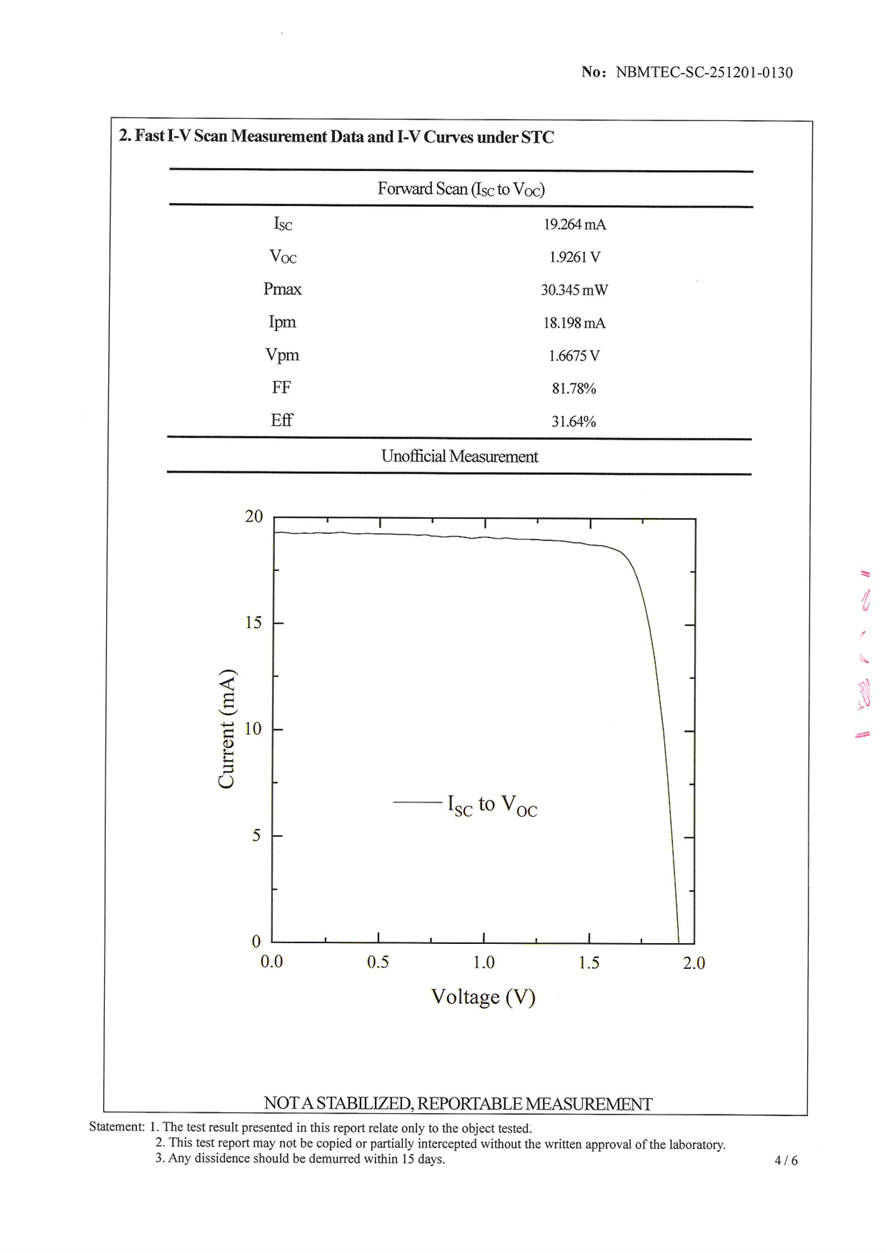

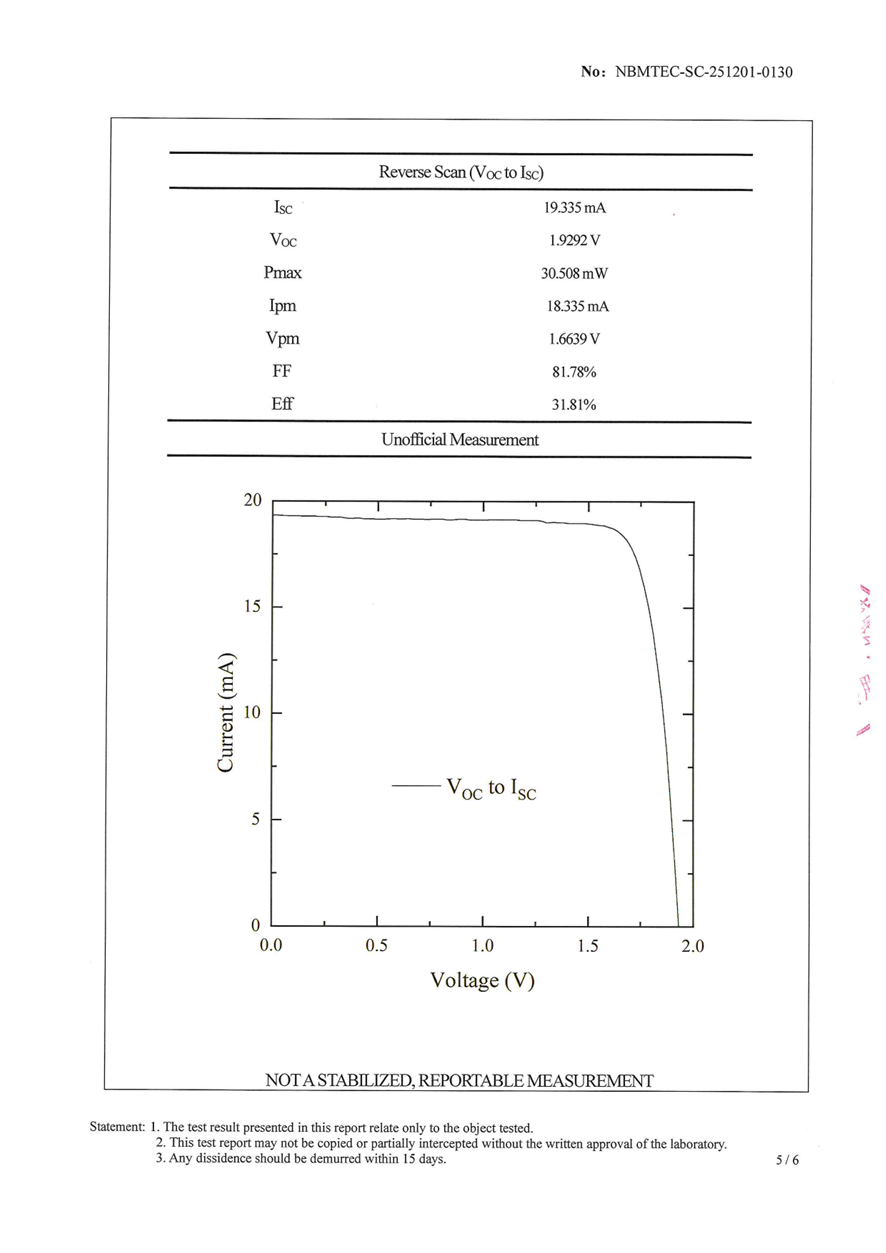

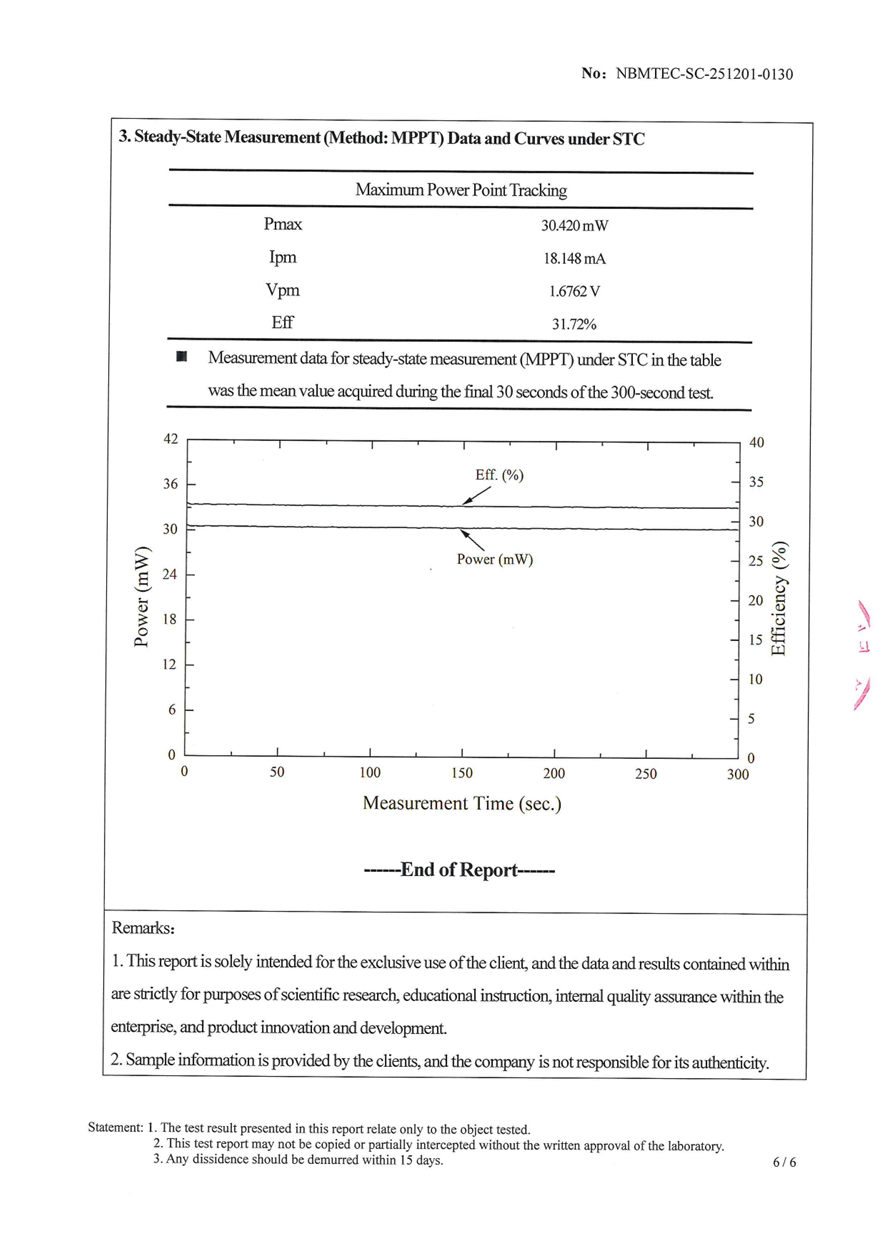


Figure S47. Certified efficiency report of the perovskite/TOPCon silicon tandem device with an area of 0.959 cm^2^ employing Me-4PACz (GuaSCN) as HTL by the AAA Steady dual light source solar simulator based on the IEC 60904-1-1: 2017 standard.

Table S1. Summarizing the surface free energies of control and target substrates.

| Units (mN.m^-1^) | Control | Target |
| --- | --- | --- |
| Surface Free Energy | 43.2 ± 6.2 | 71.2 ± 12.2 |
| Disperse Component | 38.0 ± 5.5 | 38.5 ± 6.7 |
| Polar Component | 5.2 ± 2.8 | 32.7 ± 10.2 |

Table S2. The TRPL fitted parameters of the glass/ITO/SAMs/perovskite stack by bi-exponential fitting. The incident excitation light was irradiated from the perovskite side.

|  | A_1_ | τ_1_ (ns) | A_2_ | τ_2_ (ns) | τ_ave_ (ns) |
| --- | --- | --- | --- | --- | --- |
| Control | 0.43 | 23.81 | 0.5 | 1314.71 | 1294.91 |
| Target | 0.26 | 111.23 | 0.59 | 2075.52 | 2030.20 |

Table S3. The TRPL fitted parameters of the glass/ITO/SAMs/perovskite stack by bi-exponential fitting. The incident excitation light was irradiated from glass/ITO side.

|  | A_1_ | τ_1_ (ns) | A_2_ | τ_2_ (ns) | τ_ave_ (ns) |
| --- | --- | --- | --- | --- | --- |
| Control | 0.38 | 100.21 | 0.48 | 1408.44 | 1338.68 |
| Target | 0.35 | 33.03 | 0.57 | 1853.93 | 1834.23 |

The carrier recombination lifetimes were derived from a bi-exponential fitting:

$$I\left( t \right)=I_{0}+A_{1}e^{-t/\tau_{1}}+A_{2}e^{-t/\tau_{2}}$$

The average carrier lifetime (τ_ave_) was calculated according to the following equation:

$$\tau_{ave}=\left( A_{1}\tau_{1}^{2}+A_{2}\tau_{2}^{2} \right)/\left( A_{1}\tau_{1}+A_{2}\tau_{2} \right)$$

Table S4. PbI_2_ and (100) peak intensities from GIWAXS q_z_ profiles and their intensity ratios.

| q_z_ | PbI_2_ | 100 | 100/PbI_2_ |
| --- | --- | --- | --- |
| Control | 0.098 | 0.363 | 3.72 |
| Target | 0.173 | 0.712 | 4.12 |

Table S5. PbI_2_ and (100) peak intensities from GIWAXS 2θ profiles and their intensity ratios.

| 2θ | PbI_2_ | 100 | 100/PbI_2_ |
| --- | --- | --- | --- |
| Control | 3.124 | 9.767 | 3.13 |
| Target | 3.180 | 11.422 | 3.59 |

Table S6. Comparison of *J−V* characteristics for PSCs with different structural configurations.

|  | *V*_oc_ (V) | *J*_sc_ (mA cm^-2^) | FF (%) | PCE (%) | HI (%) |
| --- | --- | --- | --- | --- | --- |
| MeO-2PACz-Forward | 1.199 | 21.42 | 82.68 | 21.23 | 1.19 |
| MeO-2PACz-Reverse | 1.197 | 21.42 | 81.74 | 20.96 |  |
| MeO+GuaSCN-Forward | 1.244 | 21.57 | 82.74 | 22.20 | 0.74 |
| MeO+GuaSCN-Reverse | 1.245 | 21.57 | 83.27 | 22.37 |  |
| 4PADCB-Forward | 1.208 | 21.45 | 81.94 | 21.22 | 1.55 |
| 4PADCB-Reverse | 1.205 | 21.44 | 80.80 | 20.89 |  |
| 4P+GuaSCN-Forward | 1.248 | 21.56 | 83.42 | 22.45 | 0.71 |
| 4P+GuaSCN-Reverse | 1.250 | 21.56 | 83.92 | 22.61 |  |

Table S7 Summary of the monolithic perovskite/silicon tandem cells based on silicon homojunction reported in literature.

| No. | published date | area  (cm^2^) | PCE  (%) | J_SC_  (mA/cm^2^) | V_OC_  (V) | FF  (%) | silicon type | polarity | reference |
| --- | --- | --- | --- | --- | --- | --- | --- | --- | --- |
| 1 | 2015/3/24 | 1 | 13.7%* | 11.5 | 1.58 | 75 | AL-BSF | n-i-p | Appl. Phys. Lett.^[1]^ |
| 2 | 2016/12/5 | 0.25 | 16.4%* | 15.3 | 1.67 | 67.6 | PERL | n-i-p | Appl. Phys. Lett.^[2]^ |
| 3 | 2017/10/13 | 1 | 22.50%* | 17.6 | 1.75 | 73.8 | PERT | n-i-p | Energy Environ. Sci.^[3]^ |
| 4 | 2018/4/16 | 1 | 16%* | 15.3 | 1.43 | 75 | AL-BSF | p-i-n | IEEE J. Photovolt.^[4]^ |
| 5 | 2018/6/25 | 4 | 20.5%* | 16.1 | 1.68 | 78 | PERT | n-i-p | Energy Environ. Sci.^[5]^ |
| 6 | 2018/8/31 | 16 | 21.8%* | 16.2 | 1.74 | 78 | PERT | n-i-p | ACS Energy Lett.^[6]^ |
| 7 | 2018/12/14 | 1 | 24.1%* | 17.8 | 1.763 | 78.1 | TOPert | n-i-p | Sci. Adv.^[7]^ |
| 8 | 2019/3/12 | 1.42 | 25.1%* | 19.5 | 1.74 | 74.7 | TOPCon^2^ | p-i-n | ACS Energy Lett.^[8]^ |
| 9 | 2019/3/19 | 0.27 | 21.1%* | 16.12 | 1.645 | 79.92 | Al-BSF | p-i-n | Nano Energy^[9]^ |
| 10 | 2019/10/8 | 4 | 23.0%* | 16.5 | 1.78 | 81 | PERT | n-i-p | ACS Energy Lett.^[10]^ |
| 11 | 2021/11/10 | 0 | 16.90% | 16.6 | 1.53 | 67 | TOPCon-BSF | n-i-p | Energies^[11]^ |
| 12 | 2022/2/8 | 1.01 | 21.34% | 17.07 | 1.8 | 69.25 | TOPerl | p-i-n | Sol. RRL^[12]^ |
| 13 | 2022/3/22 | 25 | 17.30% | 14.1 | 1.784 | 67 | TOPCon-BSF | n-i-p | ACS Appl. Energy Mater.^[13]^ |
| 14 | 2022/6/9 | 1 | 27.60% | 19.68 | 1.794 | 78.27 | PERC-TOPCon | p-i-n | Adv. Energy Mater.^[14]^ |
| 15 | 2022/8/1 | 0.25 | 23.50% | 16.74 | 1.75 | 80.21 | Al-BSF | p-i-n | Adv. Funct. Mater.^[15]^ |
| 16 | 2022/8/1 | 1.006 | 28.74%* | 19.29 | 1.907 | 78.3 | TOPerc | p-i-n | ACS Energy Lett.^[16]^ |
| 17 | 2022/10/11 | 0.124 | 28.2%* | 19.2 | 1.799 | 81.8 | TOPCon^2^ | p-i-n | Joule^[17]^ |
| 18 | 2022/10/20 | 0.1387 | 26.06%* | 18.3 | 1.72 | 82 | TOPCon^2^ | p-i-n | Sol. RRL^[18]^ |
| 19 | 2022/12/20 | 0.124 | 28.49% | 19.4 | 1.799 | 81.64 | TOPCon^2^ | p-i-n | Adv. Energy Mater^[19]^ |
| 20 | 2023/3/25 | 1 | 23.18% | 17.8 | 1.76 | 74 | TOPCon^2^ | p-i-n | Prog. Photovolt. Res. Appl.^[20]^ |
| 21 | 2023/4/15 | 0.9491 | 26.3%* | 18.73 | 1.741 | 81.2 | TOPCon^2^ | p-i-n | Nat. Commun.^[21]^ |
| 22 | 2023/4/18 | 0.132 | 29.0%* | 19.3 | 1.842 | 82.34 | TOPCon^2^ | p-i-n | Adv. Mater.^[22]^ |
| 23 | 2023/5/26 | 9 | 19% | 16.1 | 1.688 | 69.3 | TOPCon | n-i-p | Energies^[23]^ |
| 24 | 2023/8/2 | 0.25 | 23% | 16.36 | 1.75 | 80.33 | Al-BSF | p-i-n | EcoMat^[24]^ |
| 25 | 2023/8/18 | 0.25 | 23.6%* | 18 | 1.705 | 77.2 | PERC | p-i-n | Sol. Energ. Mat. Sol. C.^[25]^ |
| 26 | 2023/8/21 | 4 | 24.06% | 17.44 | 1.822 | 75.8 | PER-TOPCon | n-i-p | ACS Energy Lett.^[26]^ |
| 27 | 2023/9/26 | 4 | 28.25% | 19.13 | 1.84 | 80.55 | TOPCon^2^ | p-i-n | Energy Advances^[27]^ |
| 28 | 2023/11/9 | 0.132 | 29.20% | 19.7 | 1.83 | 81 | TOPCon^2^ | p-i-n | Nat. Energy^[28]^ |
| 29 | 2023/12/17 | 1.087 | 30.72%* | 19.58 | 1.929 | 81.54 | PER-TOPCon | p-i-n | Adv. Energy Mater.^[29]^ |
| 30 | 2024/2/4 | 0.25 | 20.88% | 15.09 | 1.746 | 79.24 | PERC | p-i-n | Prog. Photovolt. Res. Appl.^[30]^ |
| 31 | 2024/3/11 | 0.08 | 25.20% | 18.4 | 1.74 | 78.52 | TOPCon^2^ | n-i-p | Nano Energy^[31]^ |
| 32 | 2024/4/11 | 0.1 | 28.67% | 20.1 | 1.837 | 77.67 | TOPCon^2^ | p-i-n | Adv. Funct. Mater.^[32]^ |
| 33 | 2024/4/13 | 0.1344 | 23.6%* | 17.1 | 1.73 | 80 | TOPCon^2^ | p-i-n | Sci. Bull.^[33]^ |
| 34 | 2024/4/19 | 0.135 | 25.12% | 17.66 | 1.894 | 75.1 | TOPCon^2^ | n-i-p | Sol. RRL^[34]^ |
| 35 | 2024/6/5 | 1 | 16.23% | 15.41 | 1.557 | 67.65 | TOPCon-BSF | n-i-p | ACS Appl. Mater. Interfaces^[35]^ |
| 36 | 2024/6/24 | 1 | 29.4%* | 19.3 | 1.87 | 83 | TOPCon | n-i-p | J. Mater. Chem. A^[36]^ |
| 37 | 2024/7/23 | 0.13 | 30.5%* | 20 | 1.8 | 85.4 | TOPCon^2^ | p-i-n | ACS Energy Lett.^[37]^ |
| 38 | 2024/7/29 | 0.35 | 28.53% | 20.19 | 1.73 | 81.93 | TOPCon-BSF | n-i-p | Nano Energy^[38]^ |
| 39 | 2024/9/30 | 0.9226 | 28.20% | 18.82 | 1.9 | 78.91 | TOPCon^2^ | n-i-p | Nat. Commun.^[39]^ |
| 40 | 2024/10/16 | / | 27.60% | 18.1 | 1.84 | 82.9 | TOPCon^2^ | p-i-n | Mater. Today Energy^[40]^ |
| 41 | 2024/11/20 | 0.1375 | 30.2%* | 20 | 1.84 | 83.4 | TOPCon^2^ | p-i-n | Adv. Energy Mater.^[41]^ |
| 42 | 2025/1/2 | 1.0208 | 31.32% | 19.89 | 1.931 | 81.54 | PER-TOPCon | p-i-n | Adv. Mater.^[42]^ |
| 43 | 2025/2/2 | 1.035 | 30.37%* | 19.41 | 1.977 | 79.12 | PER-TOPCon | p-i-n | Adv. Funct. Mater.^[43]^ |
| 44 | 2025/3/18 | 1 | 29.8%* | 20.1 | 1.86 | 79.5 | TOPCon^2^ | p-i-n | Adv. Energy Mater.^[44]^ |
| 45 | 2025/4/8 | 1 | 26.3%* | 20.2 | 1.71 | 78 | PER-TOPCon | p-i-n | Adv. Energy Mater.^[45]^ |
| 46 | 2025/5/5 | 1 | 29.2%* | 20 | 1.86 | 79.4 | TOPCon^2^ | p-i-n | Small^[46]^ |
| 47 | 2025/5/6 | 1 | 30.4%* | 20.05 | 1.912 | 80.9 | TOPCon^2^ | p-i-n | Energy Environ. Sci.^[47]^ |
| 48 | 2025/5/27 | 0.2637 | 21.7%* | 16.2 | 1.62 | 81.3 | Al-BSF | p-i-n | Nano Converg.^[48]^ |
| 49 | 2025/5/31 | 1.1009 | 30.90% | 20.02 | 1.909 | 80.85 | PER-TOPCon | p-i-n | Sci. Bull.^[49]^ |
| 50 | 2025/6/23 | 1 | 28.26%* | 19.7 | 1.809 | 80.3 | PER-TOPCon | p-i-n | Adv. Energy Mater.^[50]^ |
| **51** | **/** | **1** | **31.7%*** | **20.55** | **1.93** | **81.08** | **TOPCon^2^** | **p-i-n** | **This work** |

*: stabilized efficiency

**Al-BSF**: Al-Back Surface Field

**TOPCon-BSF**: Tunnel Oxide Passivating Contact-Back Surface Field

**PERC**: Passivated Emitter and Rear Cell

**PERL**: Passivated Emitter and Rear Locally diffused contact

**PERT**: Passivated Emitter and Rear Totally diffused contact

**PER-TOPCon**: Passivated Emitter and Rear Tunnel Oxide Passivating Contact

**TOPerc**: Tunnel Oxide Passivating contact emitter and rear cell

**TOPerl**: Tunnel Oxide Passivating contact emitter and rear locally diffused contact

**TOPert**: Tunnel Oxide Passivating contact emitter and rear totally diffused contact

**TOPCon^2^**: Tunnel Oxide Passivating contact emitter and Rear Tunnel Oxide Passivating Contact

[1] J. P. Mailoa, C. D. Bailie, E. C. Johlin, E. T. Hoke, A. J. Akey, W. H. Nguyen, M. D. McGehee, T. Buonassisi, *Appl. Phys. Lett.* **2015**, 106.

[2] J. Werner, A. Walter, E. Rucavado, S.-J. Moon, D. Sacchetto, M. Rienaecker, R. Peibst, R. Brendel, X. Niquille, S. De Wolf, P. Löper, M. Morales-Masis, S. Nicolay, B. Niesen, C. Ballif, *Appl. Phys. Lett.* **2016**, 109.

[3] Y. Wu, D. Yan, J. Peng, T. Duong, Y. Wan, S. P. Phang, H. Shen, N. Wu, C. Barugkin, X. Fu, S. Surve, D. Grant, D. Walter, T. P. White, K. R. Catchpole, K. J. Weber, *Energy Environ. Sci.* **2017**, 10, 2472.

[4] R. L. Z. Hoye, K. A. Bush, F. Oviedo, S. E. Sofia, M. Thway, X. Li, Z. Liu, J. Jean, J. P. Mailoa, A. Osherov, F. Lin, A. F. Palmstrom, V. Bulović, M. D. McGehee, I. M. Peters, T. Buonassisi, *IEEE J. Photovoltaics* **2018**, 8, 1023.

[5] J. Zheng, C. F. J. Lau, H. Mehrvarz, F.-J. Ma, Y. Jiang, X. Deng, A. Soeriyadi, J. Kim, M. Zhang, L. Hu, X. Cui, D. S. Lee, J. Bing, Y. Cho, C. Chen, M. A. Green, S. Huang, A. W. Y. Ho-Baillie, *Energy Environ. Sci.* **2018**, 11, 2432.

[6] J. Zheng, H. Mehrvarz, F.-J. Ma, C. F. J. Lau, M. A. Green, S. Huang, A. W. Y. Ho-Baillie, *ACS Energy Lett.* **2018**, 3, 2299.

[7] H. Shen, S. T. Omelchenko, D. A. Jacobs, S. Yalamanchili, Y. Wan, D. Yan, P. Phang, T. Duong, Y. Wu, Y. Yin, C. Samundsett, J. Peng, N. Wu, T. P. White, G. G. Andersson, N. S. Lewis, K. R. Catchpole, *Sci. Adv.* **2018**, 4, eaau9711.

[8] G. Nogay, F. Sahli, J. Werner, R. Monnard, M. Boccard, M. Despeisse, F. J. Haug, Q. Jeangros, A. Ingenito, C. Ballif, *ACS Energy Lett.* **2019**, 4, 844.

[9] C. U. Kim, J. C. Yu, E. D. Jung, I. Y. Choi, W. Park, H. Lee, I. Kim, D.-K. Lee, K. K. Hong, M. H. Song, K. J. Choi, *Nano Energy* **2019**, 60, 213.

[10] J. Zheng, H. Mehrvarz, C. Liao, J. Bing, X. Cui, Y. Li, V. R. Gonçales, C. F. J. Lau, D. S. Lee, Y. Li, M. Zhang, J. Kim, Y. Cho, L. G. Caro, S. Tang, C. Chen, S. Huang, A. W. Y. Ho-Baillie, *ACS Energy Lett.* **2019**, 4, 2623.

[11] J. Hyun, K. M. Yeom, H. E. Lee, D. Kim, H.-S. Lee, J. H. Noh, Y. Kang, *Energies* **2021**, 14, 7614.

[12] S. Mariotti, K. Jäger, M. Diederich, M. S. Härtel, B. Li, K. Sveinbjörnsson, S. Kajari-Schröder, R. Peibst, S. Albrecht, L. Korte, T. Wietler, *Sol. RRL* **2022**, 6, 2101066.

[13] J. Y. Hyun, K. M. Yeom, S.-W. Lee, S. Bae, D. Choi, H. Song, D. Kang, J.-K. Hwang, W. Lee, S. Lee, Y. Kang, H.-S. Lee, J. H. Noh, D. Kim, *ACS Appl. Energy Mater.* **2022**, 5, 5449.

[14] Y. Wu, P. Zheng, J. Peng, M. Xu, Y. Chen, S. Surve, T. Lu, A. D. Bui, N. Li, W. Liang, L. Duan, B. Li, H. Shen, T. Duong, J. Yang, X. Zhang, Y. Liu, H. Jin, Q. Chen, T. White, K. Catchpole, H. Zhou, K. Weber, *Adv. Energy Mater.* **2022**, 12, 2200821.

[15] S. Lee, C. U. Kim, S. Bae, Y. Liu, Y. I. Noh, Z. Zhou, P. W. Leu, K. J. Choi, J. K. Lee, *Adv. Funct. Mater.* **2022**, 32, 2204328.

[16] K. Sveinbjörnsson, B. Li, S. Mariotti, E. Jarzembowski, L. Kegelmann, A. Wirtz, F. Frühauf, A. Weihrauch, R. Niemann, L. Korte, F. Fertig, J. W. Müller, S. Albrecht, *ACS Energy Lett.* **2022**, 7, 2654.

[17] Z. Ying, Z. Yang, J. Zheng, H. Wei, L. Chen, C. Xiao, J. Sun, C. Shou, G. Qin, J. Sheng, Y. Zeng, B. Yan, X. Yang, J. Ye, *Joule* **2022**, 6, 2644.

[18] Z. Ying, X. Yang, J. Zheng, J. Sun, J. Xiu, Y. Zhu, X. Wang, Y. Chen, X. Li, J. Sheng, C. Shou, Y. Zeng, H. Pan, J. Ye, Z. He, *Sol. RRL* **2022**, 6, 2200793.

[19] J. Zheng, H. Wei, Z. Ying, X. Yang, J. Sheng, Z. Yang, Y. Zeng, J. Ye, *Adv. Energy Mater.* **2022**, 13, 2203006.

[20] M. Singh, K. Datta, A. Amarnath, F. Wagner, Y. Zhao, G. Yang, A. Bracesco, N. Phung, D. Zhang, V. Zardetto, M. Najafi, S. C. Veenstra, G. Coletti, L. Mazzarella, M. Creatore, M. M. Wienk, R. A. J. Janssen, A. W. Weeber, M. Zeman, O. Isabella, *Prog. Photovolt. Res. Appl.* **2023**, 31, 877.

[21] X. Wang, Z. Ying, J. Zheng, X. Li, Z. Zhang, C. Xiao, Y. Chen, M. Wu, Z. Yang, J. Sun, J.-R. Xu, J. Sheng, Y. Zeng, X. Yang, G. Xing, J. Ye, *Nat. Commun.* **2023**, 14, 2166.

[22] X. Li, Z. Ying, J. Zheng, X. Wang, Y. Chen, M. Wu, C. Xiao, J. Sun, C. Shou, Z. Yang, Y. Zeng, X. Yang, J. Ye, *Adv. Mater.* **2023**, 35, 2211962.

[23] B. Marteau, T. Desrues, Q. Rafhay, A. Kaminski, S. Dubois, *Energies* **2023**, 16, 4346.

[24] E. D. Jung, C. U. Kim, Y. W. Noh, S. K. Seo, Y. I. Noh, K. J. Choi, M. H. Song, *EcoMat* **2023**, 5, e12399.

[25] N. Phung, D. Zhang, C. van Helvoirt, M. Verhage, M. Verheijen, V. Zardetto, F. Bens, C. H. L. Weijtens, L. J. Geerligs, W. M. M. Kessels, B. Macco, M. Creatore, *Sol. Energy Mater. Sol. Cells* **2023**, 261, 112498.

[26] L. Dai, S. Li, Y. Hu, J. Huang, Z. Liu, H. Shi, G. Guan, Y. Shen, B. Hu, P. R. i Cabarrocas, M. Wang, *ACS Energy Lett.* **2023**, 8, 3839.

[27] A. Walter, B. A. Kamino, S.-J. Moon, P. Wyss, J. J. Diaz Leon, C. Allebé, A. Descoeudres, S. Nicolay, C. Ballif, Q. Jeangros, A. Ingenito, *Energy Adv.* **2023**, 2, 1818.

[28] J. Zheng, Z. Ying, Z. Yang, Z. Lin, H. Wei, L. Chen, X. Yang, Y. Zeng, X. Li, J. Ye, *Nat. Energy* **2023**, 8, 1250.

[29] L. Qiao, T. Ye, T. Wang, W. Kong, R. Sun, L. Zhang, P. Wang, Z. Ge, Y. Peng, X. Zhang, M. Xu, X. Yan, J. Yang, X. Zhang, F. Zeng, L. Han, X. Yang, *Adv. Energy Mater.* **2023**, 14, 2302983.

[30] Y. Lee, C. U. Kim, Y. Woo, W.-M. Kim, J.-h. Jeong, D.-h. Kim, D.-K. Lee, K. J. Choi, I. Kim, *Prog. Photovolt. Res. Appl.* **2024**, 32, 406.

[31] Z. Che, L. Zhang, J. Shang, Y. Zhan, Y. Zhou, F. Liu, *Nano Energy* **2024**, 124, 109486.

[32] S. Jiang, Z. Ding, X. Li, L. Zhang, Z. Ying, X. Yang, Z. Yang, W. Yang, Y. Zeng, J. Ye, *Adv. Funct. Mater.* **2024**, 34, 2401900.

[33] X. Wang, J. Zheng, Z. Ying, X. Li, M. Zhang, X. Guo, S. Su, J. Sun, X. Yang, J. Ye, *Sci. Bull.* **2024**, 69, 1887.

[34] Z. Ding, Z. Liu, M. Xing, X. Xue, W. Yang, W. Liu, M. Liao, Z. Yang, Y. Zeng, J. Ye, *Sol. RRL* **2024**, 8, 2400134.

[35] D. Pyun, D. Choi, S. Bae, S.-W. Lee, H. Song, S. H. Jeong, S. Lee, J.-K. Hwang, S. Cho, H. Lee, M. Woo, Y. Lee, K. Kim, Y. Kim, C. Lee, Y. Choe, Y. Kang, D. Kim, H.-S. Lee, *ACS Appl. Mater. Interfaces* **2024**, 16, 28379.

[36] L. Duan, S. P. Phang, D. Yan, J. Stuckelberger, D. Walter, Y. Hou, W. Wang, N. Chang, A. D. Bui, A. O. Mayon, L. Chang, D. Kang, T. Duong, R. Basnet, H. Nguyen, T. White, J. Bullock, K. Weber, D. MacDonald, K. Catchpole, H. Shen, *J. Mater. Chem. A* **2024**, 12, 20006.

[37] Z. Ying, X. Guo, H. Du, X. Li, M. Zhang, Y. Zeng, X. Yang, J. Ye, *ACS Energy Lett.* **2024**, 9, 4018.

[38] C. Gao, H. Zhang, S. Ma, H. Su, H. Huang, L. He, D. Zhang, D. Du, H. Liu, W. Shen, *Nano Energy* **2024**, 129, 110066.

[39] Z. Ding, C. Kan, S. Jiang, M. Zhang, H. Zhang, W. Liu, M. Liao, Z. Yang, P. Hang, Y. Zeng, X. Yu, J. Ye, *Nat. Commun.* **2024**, 15, 8453.

[40] Z. Liu, Q. Han, Q. Wu, H. Du, M. Liao, W. Liu, Z. Yang, Y. Zeng, J. Ye, *Mater. Today Energy* **2024**, 46, 101721.

[41] X. Guo, Z. Ying, X. Li, M. Zhang, S. Su, J. Zheng, H. Du, Y. Sun, J. Wu, L. Liu, Y. Zeng, X. Yang, J. Ye, *Adv. Energy Mater.* **2024**, 15, 2403021.

[42] L. Wang, N. Wang, X. Wu, B. Liu, Q. Liu, B. Li, D. Zhang, N. Kalasariya, Y. Zhang, X. Yan, J. Wang, P. Zheng, J. Yang, H. Jin, C. Wang, L. Qian, B. Yang, Y. Wang, X. Cheng, T. Song, M. Stolterfoht, X. C. Zeng, X. Zhang, M. Xu, Y. Bai, F. Xu, C. Zhou, Z. Zhu, *Adv. Mater.* **2025**, 37, 2416150.

[43] T. Ye, L. Qiao, T. Wang, P. Wang, L. Zhang, R. Sun, W. Kong, M. Xu, X. Yan, J. Yang, X. Zhang, X. Yang, *Adv. Funct. Mater.* **2025**, 35, 2419391.

[44] L. Liu, Z. Ying, X. Li, H. Du, M. Zhang, J. Wu, Y. Sun, H. Ma, Z. He, Y. Yu, X. Guo, J. Sun, Y. Zeng, X. Yang, J. Ye, *Adv. Energy Mater.* **2025**, 15, 2405675.

[45] Y. Zhang, Y. Zhu, J. Sun, M. Hu, J. Chen, B. Duan, S. Hu, P. Hou, W. L. Tan, Z. Ku, W. Yang, J. Lu, *Adv. Energy Mater.* **2025**, 15, 2405377.

[46] Y. Sun, Z. Ying, X. Li, M. Zhang, X. Guo, H. Du, H. Li, L. Liu, J. Wu, H. Ma, Y. Yu, Z. He, Y. Zeng, X. Yang, J. Ye, *Small* **2025**, 21, 2503173.

[47] X. Liu, M. Rienäcker, M. Gholipoor, L. Fang, T. Zhao, B. Hacene, J. Petermann, R. Cai, H. Hu, T. Feeney, F. Sadegh, P. Fassl, R. Guo, U. Lemmer, R. Peibst, U. W. Paetzold, *Energy Environ. Sci.* **2025**, 18, 5599.

[48] Y. H. Jang, Y. Lee, H. S. Seo, H. Lee, K.-j. Lim, J.-K. Lee, J. Heo, I. Kim, D.-K. Lee, *Nano Convergence* **2025**, 12, 24.

[49] X. Liao, Y. Zhang, Z. Qu, Q. Zeng, M. Zhang, J. Wang, Y. Pan, J. Yang, D. Zheng, Z. Liu, L. Li, M. Xu, X. Zhang, F. Liu, *Sci. Bull.* **2025**, DOI: [10.1016/j.scib.2025.05.039](https://doi.org/10.1016/j.scib.2025.05.039).

[50] Y.-C. Wang, H.-Q. Du, C. Guo, J.-g. Wang, Q.-B. Yuan, Z.-W. Yin, Y.-Q. Lan, A.-Y. Tian, Y.-S. Xiao, W. Jiang, X.-J. Yang, Y.-X. Gao, J. Yang, X.-Y. Zhang, T. Zeng, Y.-X. Chen, W.-N. Li, M. U. Rothmann, M.-L. Xu, Y.-B. Cheng, W. Li, *Adv. Energy Mater.* **2025**, 15, 2502057.
